# The cultivation of rye in marginal Alpine environments: a comparison of the agronomic, technological, health and sanitary traits of local landraces and commercial cultivars

**DOI:** 10.3389/fpls.2023.1130543

**Published:** 2023-05-10

**Authors:** Claudia Sardella, Luca Capo, Martino Adamo, Matteo Donna, Simone Ravetto Enri, Francesca Vanara, Michele Lonati, Marco Mucciarelli, Massimo Blandino

**Affiliations:** ^1^ Department of Agricultural, Forest and Food Sciences, University of Turin, Turin, Italy; ^2^ Department of Life Sciences and Systems Biology, University of Turin, Turin, Italy

**Keywords:** landraces, marginal environments, rye, Italian Alps, agronomic parameters, bioactive compounds, technological quality, ergot sclerotia

## Abstract

Rye is a secondary crop that is characterized by a higher tolerance to climatically less favorable conditions than other cereal species. For this reason, rye was historically used as a fundamental raw material for bread production and as a supply of straw in northern parts of Europe as well as in mountain environments, such as Alpine valleys, where locally adapted landraces have continued to be cultivated over the years. In this study, rye landraces collected in different valleys in the Northwest Italian Alps have been selected as the most genetically isolated within their geographical contexts and cultivated in two different marginal Alpine environments. The traits concerning their agronomy, mycotoxin contamination, bioactive content, as well as their technological and baking quality were assessed to characterize and compare rye landraces with commercial wheat and rye cultivars. Rye cultivars showed the same grain yield level as wheat in both environments. Only the genotype selected from the Maira Valley was characterized by tall and thin culms and a proneness to lodging, thereby resulting in a lower yield capacity. Among the rye cultivars, the hybrid one presented the highest yield potential, but also the highest susceptibility to the occurrence of ergot sclerotia. However, the rye cultivars, especially the landraces, were characterized by higher concentrations of minerals, soluble fibers, and soluble phenolic acids, and thus both their flours and breads had superior antioxidant properties. A 40% substitution of refined wheat flour with whole-grain rye flour led to a higher dough water absorption and a lower stability, thereby resulting in lower loaf volumes and darker products. Agronomically and qualitatively speaking, the rye landraces diverged significantly from the conventional rye cultivars, thus reflecting their genetic distinctiveness. The landrace from the Maira Valley shared a high content in phenolic acids and good antioxidant properties with the one from the Susa Valley and, when combined with wheat flour, turned out to be the most suitable for bread making. Overall, the results have highlighted the suitability of reintroducing historic rye supply chains, based on the cultivation of local landraces in marginal environments and the production of value-added bakery goods.

## 1 Introduction

Rye (*Secale cereale* L.) is a minor cereal crop that has been cultivated for centuries in mountainous (Fracasso et al., 2022) and northern areas of Europe, where it has traditionally been used to provide flour, fodder and straw material for roof construction and basket making (Gaetano et al., 2012). The greater adoption of rye in unfavorable environments than other cereal species is due to its better adaptation to infertile and sandy soils, low temperatures, and drought conditions, which leads to a higher yield potential than that of wheat under similar conditions (Bushuk, 2001; Schittenhelm et al., 2014). Therefore, rye has spread as a crop in marginal areas, and autochthonous landraces have developed over time. Landraces are genetically heterogeneous populations generated in subsistence agriculture that have adapted to specific environmental conditions of the region where they have evolved (Camacho Villa et al., 2005). As a result of being isolated from other populations of the species, and because of the selective pressure of the local environmental factors over time, landraces are characterized by a distinct identity and good capacity to tolerate biotic and abiotic stress, and they often result in an enhanced yielding stability and intermediate yield level in low input agricultural systems (Zeven, 1998). Landraces have played a key role in the history of crops worldwide, as the practice of farmers of saving seeds from one harvest for the following sowing has promoted intra-specific diversity and enhanced genetic variation (Camacho Villa et al., 2005). The combined phenomenon of the exodus of the young workforce from mountain and rural areas, where landraces have been cultivated and thus preserved the most, has posed serious concerns about their extinction and subsequent loss of genetic diversity (de Carvalho et al., 2013). Currently, only a few rye varieties are cultivated (Persson and von Bothmer, 2002) and these are genetically less diverse and differentiated than landraces (Targońska et al., 2016). Hybrid cultivars are widespread in the growing areas of northern Europe, due to a considerable increase in grain yield linked to the heterosis phenomenon, which has been verified in intensive cropping systems (Hansen et al., 2004; Miedaner and Hübner, 2011; Laidig et al., 2017). However, crop improvement practices often utilize landrace diversity to develop new cultivars (Camacho Villa et al., 2005), particularly when the aim is to develop cultivars for marginal environments. In this regard, rye landraces are considered a source of several disease resistance genes, to share, with their wild rye ancestors, much more variation than modern cultivars, and as a useful resource for the development of sustainable crops (Newton et al., 2010). As far as the qualitative traits are concerned, rye flour has been shown to possess a high nutritional value, as it is particularly rich, just like whole-grain, in dietary fiber, including arabinoxylans, fructans, and β-glucans (Andersson et al., 2009), as well as in a wide range of bioactive compounds, such as phenolic acids, alkylresorcinols, lignans, sterols, vitamins, and minerals (Nyström et al., 2008; Andersson et al., 2014; Kulichová et al., 2019). Rye bread is typically made with different proportions of rye and wheat flour and, in the past, was considered a staple food in the marginal areas where rye was traditionally cultivated (Netting, 1981). Interest in this product has recently increased, as whole-grain rye flour can be incorporated into wheat flour in order to provide several bioactive compounds and to produce value-added baked goods (Aprodu and Banu, 2017) that meet the consumers’ growing request for functional foods (Euromonitor, 2016). However, the rheological and technological aspects of rye flour differ greatly from those of wheat flour, as its bread-making quality is mainly controlled by the carbohydrate fraction, especially by starch and arabinoxylans, and by the amylase activity, and, albeit to a lesser extent, by proteins and by the proteolytic activity (Bushuk, 2001; Buksa et al., 2010; Buksa et al., 2013). Therefore, the rheological traits and bread-making aptitude of mixes made of wheat and rye flour should be carefully evaluated in order to successfully produce landrace-based products and develop high value supply chains for marginal environments. In addition to the qualitative traits, local rye landraces are regarded as key components, from a cultural and social perspective, as the resumption of historic and traditional rye supply chains in specific environments can contribute to the recovery of abandoned and marginal areas where rye was a typical agricultural product in the past (Leoni et al., 2021). The genetic variation and diversity of landraces originating from the northern and eastern parts of Europe, which are characterized by cold winters, as well as the genetic distances and relationships among landraces and between landraces and commonly cultivated improved varieties have already been investigated (Kühn and Hammer, 1979; Persson and von Bothmer, 2002; Larsson et al., 2019). A few studies (Peratoner et al., 2016; Leoni et al., 2021; Colombo et al., 2022) have recently agronomically and/or qualitatively characterized and compared modern populations of rye cultivars with landraces collected from farmers in the central Italian Alpine valleys, thereby highlighting the current interest in restoring the cultivation of traditional cereal crops, such as rye, and their supply chains. The reconversion of abandoned farmlands into productive areas through farming can in fact positively affect the access to and relaunching of marginal areas. Nevertheless, to the best of the authors’ knowledge, no study that takes into account a wide multiplicity of agronomic and qualitative parameters has been published about the cultivation of rye landraces in marginal Alpine environments. A previous study (Adamo et al., 2021) examined the genetic diversity and structure of rye landraces sampled in different valleys in the Northwest Italian Alps to understand which ones deserved conservation efforts, due to their authentic adaptation to the environmental conditions at their site of origin. In the present work, we have selected three out of the 16 analyzed landraces that have shown the highest genetic distance and we have carried out field experiments in two different marginal Alpine environments over two growing seasons. The landraces were characterized, from an agronomic, technological, and qualitative point of view, according to the low-input cropping system in force in the growing areas. One wheat and two modern rye cultivars, which were used as controls, were cultivated side by side and investigated along with the rye landraces.

## 2 Material and methods

### 2.1 Experimental design

Field experiments were carried out in the 2019-20 and 2020-21 growing seasons in a hilly location (Bruzolo, TO; 45° 08’ N, 7° 11’ E; altitude 450 m a.s.l., in the Susa Valley) and in a mountain location (Entracque, CN; 44° 14’ N, 7° 23’ E; altitude 900 m a.s.l., in the Gesso Valley), both of which are located in Alpine valleys in Northwest Italy, where rye was traditionally cultivated for the production of flour. The position of the Susa Valley, that is, at the center of the Alps, determines less rainfall than other Northwest Italian valleys; it has mild winters and generally dry springs and summers, and a high frequency of windy days. The experiment was carried out on the south-facing side of the central sector of the hilly valley in a shallow alluvial soil of medium fertility. Since the territory is surrounded by high mountains, the climate in the Gesso Valley mountains is characterized by cold winters and hot summers, although it is not far from the Mediterranean Sea. The trial was set up in deep and shallow soil, originating from a glacial deposit. The locations where the field experiments were located are remarkably different in terms of ecological conditions: the climate in Bruzolo is sub-Mediterranean, while Entracque is characterized by a hypomesoxeric climate, according to Gaussen-Bagnouls classification method (Cagnazzi and Marchisio, 1998), with an average annual air temperature of 12 °C and 10 °C and an average annual precipitation of 792 mm and 1107 mm in Bruzolo and Entracque, respectively, considering 19 years of observations (2002-2021; ARPA Piemonte agrometeorological service).

Five rye genotypes were sown in each location, together with an ordinary bread-making wheat (cv. Solehio, Agroalimentare Sud Spa, Melfi, Italy), which was used as a control. The three compared local rye landrace genotypes, called Susa, Maira and Tanaro, according to the respective Valleys of origin, were identified during a survey carried out in the alpine growing areas of Northwest Italy by Adamo et al. (2021) and selected as the genotypes with the highest genetic distances. The rye landraces originating from the Maira Valley and from the Susa Valley showed high values of nucleotide diversity and resulted to be the most genetically isolated within this geographical context. The rye landraces were compared with a commercial population rye cultivar (cv. Antoninskie, with Poznańska Hodowla Roślin Sp. z.o.o., Tulce, Poland, being responsible for its selection and conservation, and the Società Italiana Sementi, San Lazzaro di Savena, Italy for its supply), and with a hybrid rye cultivar (cv. Su Nasri, with Saaten-Union GmbH, Isernhagen, Germany, being responsible for its selection and conservation, and RV Venturoli, Pianoro, Italy for its supply), both of which are widely cultivated in the growing areas of north Europe and available on the Italian market.

The cereal genotypes from each location were assigned to experimental units using a completely randomized block design, with three replicates. The plot was 12 m^2^ (6 x 2 m) in size. The previous crop in each growing season had been bread wheat or a meadow in the hilly and mountain locations, respectively, and the latter was often also used for grazing. The plots were seeded after an autumn ploughing (30 cm) and disk harrowing to prepare a suitable seedbed. Mechanical sowing was conducted in 12 cm wide rows (on October 30 in both 2019 and 2020 at Bruzolo, and on October 18 and October 5 in 2019 and 2020, respectively, at Entracque) at a seeding rate of 350 seeds m^-2^, except for the hybrid rye for which a seeding rate of 200 seeds m^-2^ was applied. According to the low-input cropping system in force in the growing areas, no mineral or organic fertilizations, or chemical and mechanical treatments were applied to control weeds, diseases or insects during the cultivation.

### 2.2 Agronomic and yield parameters

The full flowering date, that is, when 50% of the ears had reached the complete extrusion of anthers from the spikelets (growth stage, GS 65, according to the BBCH scale), was monitored during spring and expressed as days from April 1^st^, while the flowering duration was counted considering the days that had elapsed between the first release of the anthers and complete anthesis (GS 60-69). The number of ears per unit of grown area (ear density) was counted manually before the harvest in two sampling areas (625 cm^2^) per plot. The number of kernels per ear was calculated in 10 randomly selected ears from each sampling area. The average plant height and the percentage of plot with severe lodging were recorded for each plot. The culm diameter was measured on the 1^st^ internode length in 10 plants per plot using a digital gauge.

The plots were harvested (on July 14 and July 23 in Bruzolo 2020 and 2021, and on August 6 and August 12 in Entracque 2020 and 2021, respectively), by means of a Walter Wintersteiger cereal plot combine-harvester, and the straw was collected behind the combine-harvester and weighed immediately after harvesting. The grain yield (GY) and straw yield results were expressed on a dry weight (DW) basis, and the harvest index (HI) values were obtained by dividing the grain yield by the aboveground biomass (grain + straw) of each sample, expressed as a percentage. Aliquots of 2 kg of grain were taken from each plot, to determine the test weight (TW) and the grain moisture content using a GAC^®^ 2000 Grain Analyzer (Dickens-John Auburn, IL, USA). The thousand-kernel weight (TKW) was determined on two 200-kernel sets for each sample using an electronic balance.

### 2.3 Sanitary risk

The number of ergot sclerotia were visually counted in 500 g grain samples randomly collected from each plot and expressed as g of sclerotia per kg of grain, according to the Commission Regulation (EU) 2021/1399 of 24 August 2021 (European Commission, 2021). The deoxynivalenol (DON) concentration was determined in whole-grain flour, using the ELISA method, by means of RIDASCREEN^®^ DON direct competitive immunoassays (R-Biopharm, Darmstadt, Germany), according to the method reported by Nguyen et al. (2019).

### 2.4 Chemical analyses

#### 2.4.1 Chemicals

2-2′-Azino-bis(3-ethylbeenzothiazoline-6-sulfonic acid) diammonium salt (ABTS), ethanol (CHROMASOLV^®^, 99.8%), ethylacetate (CHROMASOLV^®^, 99.8%), (±)-6-hydroxy-2,5,7,8-tetramethylchromane-2-carboxylic acid (Trolox, 97%), hydrochloric acid (HCl, 37.0%), Iron (III) chloride hexahydrate (FeCl_3_*6H_2_O, ≥ 98.0%), methanol (CHROMASOLV^®^, 99.9%), potassium sulfate (K_2_SO_4_, ≥ 99.0%), sodium hydroxide (NaOH, ≥ 98.0%), 2,4,6-Tris(2-pyridyl)-s-triazine (TPTZ), trifluoroacetic acid (TFA), sodium carbonate (Na_2_CO_3_) and phenolic acid standards (caffeic acid ≥ 98%, *p*-coumaric acid ≥ 98%, *t*-ferulic acid ≥ 99%, gallic acid ≥ 99%, protocatechuic acid ≥ 99%, *p*-hydroxybenzoic acid ≥ 99%, sinapic acid ≥ 98%, syringic acid ≥ 95% and vanillic acid ≥ 97%) were purchased from Sigma-Aldrich (St. Louis, Missouri, USA). Monosaccharide standards (L(+) Arabinose and D(+) Xylose) were purchased from Extrasynthese (Genay, France). Water (HPLC grade) was obtained from ELGA PURELAB Ultra system (M-medical, Cornaredo, Milan, Italy).

#### 2.4.2 Proximate composition analyses

The harvested grains were mixed thoroughly, and a 2 kg grain sample was taken from each plot, while representative sub-samples (500 g) were ground to whole-meal using a laboratory centrifugal mill equipped with a 1-mm sieve (Model ZM-200, Retsch, Haan, Germany). The grain protein content (GPC) and the ash content were determined according to AACC method 39-10.01 (AACC International, 2008) on whole-grain flour, using an NIRsystems 6500 monochromator instrument (Foss-NIRsystems, Laurel, MD, USA). The samples were ground to a fine powder (particle size < 500 μm) with a Cyclotec 1093 sample mill (Foss, Padua, Italy), and stored at -25 °C, prior to the chemical analyses. The moisture content, which was determined to express the results on a DW basis, was obtained by oven-drying the < 500 μm samples at 105 °C for 24 h. The β-glucan determination was performed using a Megazyme mixed-linkage β-glucan assay kit, according to the producer’s instructions (Megazyme International Ireland Ltd, Wicklow, Ireland). The total dietary fiber (TDF) was determined according to the AOAC 985.29-1986 enzymatic-gravimetric method (AOAC International, 2003) on a mixed representative sample for each genotype. All the samples were analyzed in triplicate, except for the TDF, which was instead analyzed once for each genotype, and the results are presented as DW%.

##### 2.4.2.1 Determination of the total arabinoxylans (TAX)

The monosaccharide composition of the TAX was determined by means of high-performance liquid chromatography with reflectance index detection (HPLC/RID) after acid hydrolysis, according to the procedure proposed by Buksa et al. (2012), albeit with some modifications. Aliquots of 0.5 mL of deionized water and 2 mL of 2 M TFA were added to 25 mg of each sample, and the mixtures were hydrolyzed for 3 h at 90 °C in a water bath. The samples were vortexed every 30 min during hydrolysis to aid dispersion. The samples were then cooled and subsequently centrifuged at 3300 rpm for 10 min. Aliquots (2 mL) of the clear supernatants were collected, to which 8 mL of bidistilled water was added. The samples were neutralized with dry Na_2_CO_3_ to pH 7, filtered using a syringe filter (pore diameter 0.20 µm) and then 10 µL of the sample was injected into an HPLC system, Agilent 1200 Series (Agilent Technologies, Santa Clara, CA, USA) coupled with an Agilent 1200 Series RI detector. Separations were carried out using SecurityGuard Cartridges, Carbo-Pb (4 x 3.0 mm, Phenomenex) and an SP08010 column (Shodex); the column temperature was set at 70 °C and the mobile phase was bidistillated water (flow rate of 0.6 mL min^-1^). Arabinose and xylose were identified using the retention times and the reflectance indexes of their respective standards, and the TAX content was calculated as the sum of the two sugars. Solutions of individual monosaccharide standards (L(+) Arabinose and D(+) Xylose) were prepared and diluted to different concentrations to obtain calibration curves for quantification purposes. All the samples were analyzed in triplicate, and the results are presented as DW%. The degree of arabinose substitution to the xylose units (A/X ratio) was calculated by dividing the arabinose content by the xylose content.

#### 2.4.3 Determination of the phenolic acid composition and antioxidant capacity (AC)

Soluble (free and conjugated, SPAs) and cell wall-bound phenolic acids (CWBPAs) were extracted and quantified according to the procedure described in detail by Giordano et al. (2019). Briefly, SPAs were extracted twice with an 80:20 (v/v) ethanol:water solution. The supernatants were collected and then evaporated to dryness under a nitrogen stream. Samples were hydrolyzed with 2 M NaOH (400 μL) for 2 h under continuous stirring at 4 °C. After acidification to pH 2 with HCl, the soluble phenolic acids were extracted three times with 500 μL of ethyl acetate. For the extraction of CWBPAs, the samples were extracted as described before in order to remove the soluble phenolic acids. The remaining pellet was hydrolyzed for 4 h under continuous stirring at 4 °C, by adding 2 M NaOH (400 μL). After acidification to pH 2 with HCl, the bound phenolic acids were extracted twice with 800 μL of ethyl acetate and then centrifuged. The phenolic extracts were filtered through a 0.2 μm filter and then analyzed by means of a reverse phase high-performance liquid chromatograph Agilent 1200 Series (Agilent Technologies, Santa Clara, CA, USA) coupled with an Agilent 1200 Series diode array detector (RP-HPLC/DAD). Separations were carried out using a 150 x 4.6 mm, 5 μm, Gemini RP-18 column (Phenomenex, Torrance, CA, USA); the column temperature was set at 35 °C. Phenolic acids were identified using the retention times and the UV/Vis spectra of their respective standards.

The AC of the whole-grain flour and bread samples was determined by means of both ABTS (AC_ABTS_) and ferric reducing antioxidant power (AC_FRAP_) assays, following the QUENCHER approach (direct measurement of AC on solid samples) previously described by Serpen et al. (2012). Three individual extractions were carried out for each sample (*n* = 3), and the results are expressed as mg kg^-1^ (DW) for SPAs and CWBPAs, and as mmol Trolox equivalents (TE) kg^-1^ of sample (DW) for AC.

### 2.5 Technological quality analyses

The technological characterization was carried out by employing whole-grain flour blends of both bread wheat and the rye varieties with refined wheat flour in a proportion of 40:60, according to the conventional commercial use of rye flour for baking in the growing areas. The refined wheat flour was used on its own as a control. The whole-grain wheat and rye flours were obtained by completely grinding the harvested grains, using a Retsch ZM 200 (Retsch GmbH, Haan, Germany) fitted with a 1-mm aperture sieve. The refined wheat flour was supplied by a milling company (0.76% ash content) and the main rheological properties are reported in **Table S1**.

#### 2.5.1 Rheological properties of the flour

The mixing and pasting behaviors of the refined control and of the whole-grain wheat and rye flour blends were studied using a Mixolab^®^ analyzer (Chopin Technologies, Villeneuve la Garenne, France), according to the ICC Standard Method 173 (ICC, 2011). This instrument allows specific information to be obtained about the behavior of dough constituents (starch, protein, water) by continuously measuring the torque (Nm) produced by the passage of the dough between two mixing blades submitted to both shear stress and temperature changes. The resulting Mixolab^®^ curve is separated into five different phases. A detailed description of the physical changes that occur during a Mixolab^®^ measurement was reported in Rosell et al. (2007). Analyses were carried out through two different approaches. According to the standard ‘Chopin+’ protocol, an adapted water supply was used to quantify the water absorption capacity (WA, %) and the dough development time (DDT, min) of each of the flour blends, which represent the amount of water that flour can absorb and the time necessary to reach a pre-set reference dough consistency and to produce a torque (peak C1) of 1.1 Nm ± 0.05 at 30 °C. A faster Mixolab^®^ mixing protocol, called FAST protocol, was then applied at a constant WA of 56%, as described by Dubat and Lebrun (2019), albeit with a few modifications of the assay conditions. The starting temperature was set at 45 °C and the heating cycle, which was initiated after 4 min, was set between 45 and 90 °C, while the cooling cycle, which was initiated after 5 min, was set between 90 and 50 °C. The heating and cooling rates were set at 8 °C min^-1^. The following parameters, which were derived from the curve, were obtained: DDT, or the time necessary to reach the maximum torque (C1) at 45 °C; dough stability (DS, min), or the elapsed time at which the produced torque is kept at the C1 torque value ( ± 11%); thermal weakening, i.e. decrease in dough consistency due to mechanical shear stress and a temperature increase from 45 to 60 °C (C1-C2, Nm), which provides information about the protein quality; starch gelatinization, i.e. an increase in viscosity due to the absorption of water by the starch granules and an increase in temperature from 60 to 90 °C (C3-C2, Nm); cooking stability, i.e. the stability of the hot gel at a constant temperature of 90 °C (C3-C4, Nm); and cooling setback, i.e. the retrogradation of the starch in the cooling phase from 90 to 50 °C (C5-C4, Nm).

The solvent retention capacity (SRC) test was adopted to provide information on the water adsorption and on the content of pentosans, according to AACC method 56-11.02 (AACC International, 2008). The following solvents were tested separately: water (W-SRC) and 50% sucrose (S-SRC). Briefly, deionized water or a 50% sucrose solution was added to 5 g of a flour sample, in different centrifuge tubes. After 20 min of hydration and a periodic vigorous shaking (every 5 min for 5 s), the samples were centrifuged at 1000×g for 15 min. The supernatant was decanted, and the pellet was weighed. The quantity of the absorbed solutions is expressed as % SRC.

#### 2.5.2 Bread-making procedure and bread characterization

The twelve mixes (one with whole-grain wheat and five with whole-grain rye flours blended with refined flour in the proportion 40:60, from each experiment) and the refined flour on its own were employed to prepare bread in a bread-making machine. The whole-grain flours of the wheat and rye genotypes were both collected from field experiments carried out in the 2019-20 growing season and milled as previously reported. Flour (1000 g) was added to water (670 g), as were 2.5 g of powder yeast and 2 g of salt. After 13 min of kneading, the dough was manually divided into three loaves of 550 g each and left to rest for 15 min at room temperature. Leaving was then carried out for 50 min at 30 °C and at 70% humidity. The loaves were baked for 40 min at 235 °C in a bakery oven. The specific volume, color, moisture content and antioxidant capacity of each loaf of bread were determined. The loaf volume was determined 1 h after baking, by means of rapeseed displacement, according to AACC method 10-05.01 (AACC International, 2008). The chromatic characteristics of the bread crust and crumbs were determined using a Minolta Chroma Meter reflectance spectrophotometer (Model CR-400, Minolta Co., Osaka, Japan). Standard illuminant C was used as the reference. The analysis was performed in triplicate at three different points for each loaf. The lightness (L*), redness (a*) and yellowness (b*) color values were determined directly by the instrument, in accordance with Commission Internationale de l’Éclairage (CIE, 1986). The total color difference, that is, the Δ*E* value of the bread slices from the refined control, was calculated according to Mohd Jusoh et al. (2009).

Bread samples were freeze-dried using a Lyovapor L-200 Freeze Dryer (Buchi, Flawil, Switzerland) and the lyophilized samples were ground to a fine powder using a Cyclotec 1093 sample mill. The moisture content was obtained by oven-drying the samples at 105 °C for 24 h and the AC was determined by conducting the same assays that had been performed on the whole-grain samples (AC_ABTS_ and AC_FRAP_). The AC results are expressed as mmol TE kg^-1^ (DW).

### 2.6 Statistical analyses

The data were compared separately for the two different Alpine environments, by means of an analysis of variance (ANOVA), to evaluate the effect of genotype (G), harvest year (Y) and their interaction (G × Y) on the agronomic and sanitary traits, the qualitative parameters, and the phytochemical content and profile of the whole-grain flours obtained from the wheat and rye varieties. The effect of bread formulation (BF) and Y and their interaction (BF × Y) on the rheological properties of the twelve blends and the refined control flour was also investigated separately for the two environments. One-way ANOVA was performed on samples from 2020 harvest to evaluate the effect of BF on the bread-making aptitude and the antioxidant properties of the composite breads. Means were identified as significantly different (*p* ≤ 0.05) based on the Ryan/Einot and Gabriel/Welsch (REGW-F) statistical test. Prior to the analysis, data were tested for normality and homoscedasticity. Pearson correlation analysis was performed to investigate the relationships between the agronomic, nutritional and phytochemical, qualitative and rheological traits. Statistical analyses were carried out by means of SPSS for Windows statistical package, version 28.0 (SPSS, Inc., Chicago, IL, USA).

Genetic data were obtained using a ddRAD (double digest Restriction Associated DNA) sequencing approach. Libraries were produced according to the IGATech (Udine, Italy) custom protocol, with minor modifications with respect to Peterson et al. (2012). The adopted raw data clean-up and preliminary analysis procedures are described in Adamo et al. (2021). Nucleotide diversity (π) was calculated in the Stacks 2.0 environment (Catchen et al., 2011). The genetic distances between the rye cultivars were calculated as Prevosti’s distances between the populations (Prevosti et al., 1975) in the R environment with the *prevost.dist* function of the R package, ‘poppr’ v 2.9.3 (Kamvar et al., 2015) and visualized by means of a neighbor joining tree (nj). The tree topologies were sampled with a 1000 bootstrap, and a minimum bootstrap value of 75 was considered as significant.

Agronomic and qualitative parameters were used to cluster the rye cultivars into the main groups using a similarity approach. The most representative variables were selected by experts: culm diameter, plant height, HI, GY and TKW were selected to describe the variability of the agronomic parameters, while TW, GPC, β-glucans, TAX, SPAs, CWBPAs, DS, thermal weakening (C1-C2) and ergot sclerotia were selected to describe the variability of the quality parameters. A cluster analysis was performed, in the R environment, using the R package ‘factoextra’ v1.0.7 (Kassambara and Mundt, 2020). A preliminary principal component analysis (PCA) was performed to determine the influence of the variables on the cultivar ordination. The most probable number of clusters was predicted, using both the silhouette and the wss (or elbow) methods, and hierarchical clustering dendrograms were then prepared, using Ward’s minimum variance method (Ward, 1963), to describe the Euclidean distance of the cultivars.

## 3 Results

### 3.1 Meteorological trends

The two growing seasons showed different meteorological trends throughout the cereal crop cycles (**Table S2**). The precipitations in the 2019-20 growing season were 298 and 494 mm greater than in the 2020-21 season in the hilly and mountain environment, respectively. The difference in rainfall was mainly concentrated in the tillering and stem elongation stages (March to May). The growing degree days (GDDs) were higher (on average +8 °C day^-1^) from April to June in 2019-20 than in 2020-21. As expected, the rainfall and temperatures were higher and lower, respectively, in the mountain environment than in the hilly one.

### 3.2 Agronomic parameters

On average, the GY was higher in the hilly environment (4.4 t ha^-1^) than in the mountain one (3.3 t ha^-1^), due to an overall higher number of kernels per ear in 2020, and ear density and TKW in 2021 (**Table 1**). ANOVA did not show any significant differences between the GY of the rye genotypes and bread wheat in either site, except for Maira. Overall, the rye genotypes had the same ear density as wheat, except for the hybrid sown at a lower seeding rate in the mountain environment, but they resulted in a higher number of kernels per ear and in a lower TKW, although TKW only led to significant differences in the hilly site. As expected, the rye genotypes showed a higher straw yield (hilly site +52%) and plant height (+94% and +89% in the hilly and mountain environments, respectively, **Table S3**) than wheat, which resulted in a higher HI (+26%) in the hilly site. Moreover, wheat had the same precocity in the mountain site as most of the rye genotypes, but a shorter duration of flowering (**Table S3**). As far as the rye genotype comparison is concerned, the hybrid resulted in the highest GY, although this difference was not significant. The heterosis effect of this cultivar resulted in a good tillering ability, thus leading to a complete recovery of the ear density in the hilly site, and to the highest culm diameter in both environments, compared to the other genotypes. Moreover, the hybrid had a lower plant height and a higher HI than the other rye genotypes.

**Table 1:** Effect of the genotype, the harvest year and their interaction on the agronomic traits^a^ of the investigated wheat and rye cultivars, cultivated in two different marginal Alpine environments.

Environment	Factors	Source of variation	GY (t ha^-1^)		Ear density (n° m^-2^)		Kernels ear^-1^ (n°)		TKW (g)		Straw yield (t ha^-1^)		HI (%)	
Hilly (450 m)	**Genotype (G)**	Bread wheat	4.7	a	436	a	43	b	47.5	a	5.7	b	45.4	a
		Susa rye	4.8	a	388	a	57	ab	32.7	c	9.6	a	34.6	c
		Maira rye	2.6	b	377	a	59	a	27.7	d	7.9	a	24.5	d
		Tanaro rye	4.8	a	352	a	60	a	34.3	bc	9.6	a	34.1	c
		Commercial rye	4.0	a	323	a	69	a	37.1	b	8.2	a	33.9	c
		Hybrid rye	5.2	a	320	a	67	a	36.2	b	8.3	a	39.8	b
		*p* (F)	***		ns		***		***		***		***	
	**Year (Y)**	2020	4.0	b	361	a	56	a	37.4	a	10.1	a	28.5	b
		2021	4.7	a	371	a	62	a	34.4	b	6.4	b	42.3	a
		*p* (F)	**		ns		ns		***		***		***	
	**G × Y**	*p* (F)	ns		ns		*		*		ns		ns	
Mountain (900 m)	**Genotype (G)**	Bread wheat	3.0	ab	473	a	34	b	40.5	a	6.3	a	33.3	ab
		Susa rye	3.4	a	387	ab	55	a	36.4	a	6.9	a	33.6	ab
		Maira rye	2.2	b	412	ab	53	a	31.2	a	6.6	a	26.2	c
		Tanaro rye	3.7	a	347	ab	56	a	33.8	a	6.9	a	37.3	ab
		Commercial rye	3.2	ab	323	ab	53	a	34.9	a	7.5	a	31.7	b
		Hybrid rye	4.0	a	301	b	59	a	34.1	a	7.5	a	38.1	a
		*p* (F)	**		**		***		ns		ns		***	
	**Year (Y)**	2020	3.2	a	416	a	44	b	37.6	a	9.0	a	26.3	b
		2021	3.3	a	332	b	60	a	32.6	b	4.9	b	40.5	a
		*p* (F)	ns		*		***		**		***		***	
	**G × Y**	*p* (F)	ns		ns		ns		ns		ns		ns	

a GY, grain yield; TKW, thousand kernel weight; HI, harvest index. The results are expressed on a DW basis. Means followed by different letters are significantly different, according to the REGW-F test [(*) *p* (F) ≤ 0.05, (**) *p* (F) ≤ 0.01, (***) *p* (F) ≤ 0.001, and ns, non-significant].

Maira showed the greatest differences in the agronomical and yield traits in the landrace ryes: this genotype resulted in a lower GY (-73%) in both environments, as a consequence of the lower TKW; moreover, it had the longest flowering duration, the lowest HI, the greatest plant height and the thinnest culm diameter. These morphological traits led to an extensive lodging phenomenon in both years, especially in the hilly environment, which was not observed for the other genotypes. Susa and Tanaro reported very similar agronomical traits to the commercial population cultivar.

Overall, the GY was higher in 2021 than in 2020 in the hilly site, while an opposite trend was observed for the straw yield in both locations, although the G × Y interaction was never significant.

### 3.3 Ergot sclerotia contamination and DON contamination

**Figure 1:**
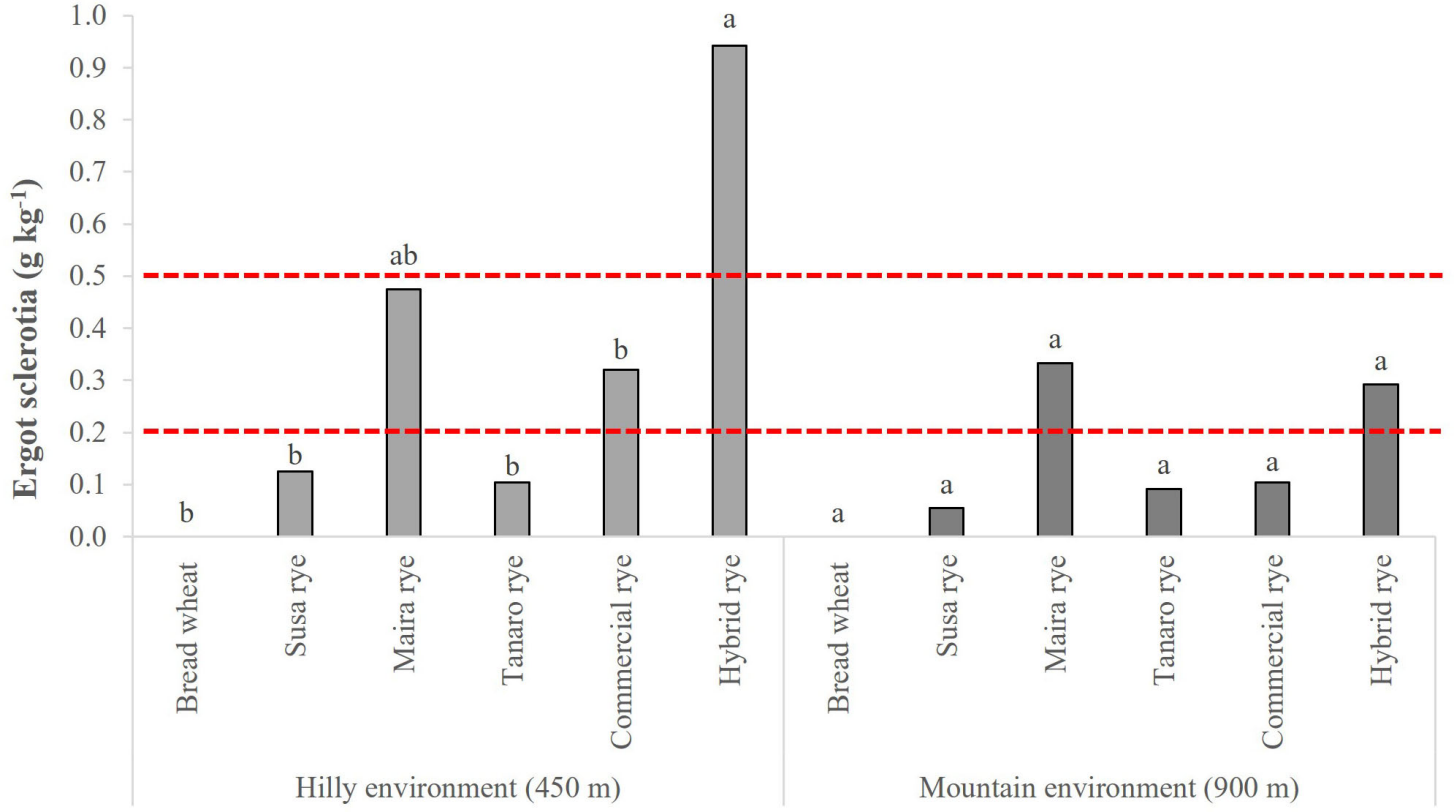
Comparison of ergot sclerotia contamination of the investigated wheat and rye genotypes. The data are averaged over 2 years and for 3 replications. Different letters on bars indicate significant differences [*p* (F) ≤ 0.05]. The 0.5 g kg^-1^ limit represents the maximum level until 30^th^ June 2024, according to Reg. (EC) No. 1881/2006 (European Commission, 2006); the 0.2 g kg^-1^ limit represents the maximum level from 1^st^ July 2024, according to Reg. (EU) No. 2021/1399 (European Commission, 2021).

Sclerotia of *Claviceps purpurea* (Fr.) Tul. were found in both sites and in both years among the collected samples of the rye genotypes, but they were never detected among those of the bread wheat (**Figure 1**). On average, the occurrence of ergot sclerotia was higher in the hilly environment in 2020 than in 2021, while no difference was reported between the years at the mountain site, which reported a lower risk than the hilly one. A significantly higher ergot sclerotia content was observed for the hybrid rye in the hilly environment than all the other genotypes, although Maira did not differ significantly from the hybrid rye. Furthermore, the sclerotia per grain in the hybrid cultivar and in Maira exceeded the legal limit (EU No. 2021/1399) at both sites, as well as in the commercial population cultivar in the hilly site. The DON content was under the limit of detection in all of the wheat and rye samples.

### 3.4 Qualitative kernel traits and compositional analyses of the whole-grain flour

Two-way ANOVA showed significant effects of the genotype factor on all the investigated qualitative traits of the grain and of the whole-grain flour in both growing environments, except for the calculated A/X ratio, which was in the same range for wheat and rye (**Table 2**). The average values of TW for the rye samples were -1.2 and -3.8% lower than for the wheat control, in the hilly and mountain sites, respectively. However, a great variation of the TW trait was observed among the rye varieties, with Susa showing the highest value in both environments, followed by Tanaro, the hybrid rye, the commercial rye and Maira. As a consequence of the lower TW, Maira recorded the highest ash content in both environments, which was significantly higher than those of the hybrid rye and the wheat control (+8 and +7% in the hilly and mountain sites, respectively), although it did not differ significantly from those of the other two landraces and the commercial rye. On average, the highest ash content was observed in the rye varieties (+4 and +3%, compared to wheat, in the hilly and mountain sites, respectively) and in the mountain environment (+2% with respect to the hilly site), which was also characterized by higher GPCs than the hilly one (on average +4%). Moreover, Maira registered the highest GPC of all the investigated rye cultivars in both sites, but did not differ significantly from the wheat control (**Table 2**).

**Table 2:** Effect of the genotype, the harvest year and their interaction on the qualitative kernel parameters and compositional traits^a^ of whole-grain flour of the investigated wheat and rye cultivars, cultivated in two different marginal Alpine environments.

Environment	Factors	Source of variation	TW (kg hL^-1^)		Ash (%)		GPC (%)		TAX (%)		A/X		β-glucans (%)		TDF (%)
Hilly (450 m)	**Genotype (G)**	Bread wheat	75.1	ab	1.89	c	10.1	ab	6.1	b	0.78	a	0.9	b	15.2
		Susa rye	76.0	a	1.94	abc	9.1	c	8.9	a	0.74	a	2.2	a	15.7
		Maira rye	71.8	c	2.04	a	10.6	a	8.7	a	0.74	a	2.0	a	15.7
		Tanaro rye	74.4	ab	1.99	abc	9.2	c	8.6	a	0.72	a	2.1	a	15.4
		Commercial rye	73.6	bc	1.99	ab	9.5	bc	8.5	a	0.78	a	2.1	a	15.5
		Hybrid rye	75.0	ab	1.91	bc	9.1	c	8.5	a	0.82	a	2.1	a	16.7
		*p* (F)	***		***		***		***		ns		***		
	**Year (Y)**	2020	75.4	a	1.87	b	9.7	a	8.2	a	0.80	a	1.9	a	14.6
		2021	73.3	b	2.05	a	9.5	a	8.3	a	0.73	a	1.9	a	15.8
		*p* (F)	***		***		ns		ns		ns		ns		
	**G × Y**	*p* (F)	ns		*		ns		ns		ns		ns		
Mountain (900 m)	**Genotype (G)**	Bread wheat	76.6	a	1.95	bc	11.3	a	6.4	b	0.86	a	0.8	c	15.0
		Susa rye	75.2	ab	1.99	ab	9.3	b	8.8	a	0.75	a	2.1	a	16.9
		Maira rye	72.8	c	2.08	a	11.1	a	8.4	a	0.74	a	2.0	ab	16.1
		Tanaro rye	73.9	bc	2.00	ab	9.4	b	8.7	a	0.81	a	2.1	ab	15.3
		Commercial rye	73.0	c	1.97	ab	9.6	b	8.5	a	0.75	a	1.9	b	14.4
		Hybrid rye	73.5	bc	1.96	b	9.3	b	8.6	a	0.77	a	2.0	ab	16.3
		*p* (F)	***		*		***		***		ns		***		
	**Year (Y)**	2020	74.2	a	2.03	a	10.1	a	8.3	a	0.81	a	1.9	a	16.8
		2021	74.1	a	1.96	b	9.9	a	8.1	a	0.75	b	1.8	b	15.6
		*p* (F)	ns		**		ns		ns		*		*		
	**G × Y**	*p* (F)	ns		ns		ns		ns		ns		**		

a TW, test weight; GPC, grain protein content; TAX, total arabinoxylans; A/X, arabinose to xylose ratio; TDF, total dietary fiber. The results are expressed on a DW basis. Means followed by different letters are significantly different, according to the REGW-F test [(*) *p* (F) ≤ 0.05, (**) *p* (F) ≤ 0.01, (***) *p* (F) ≤ 0.001, and ns, non-significant].

The TAX content was found to be significantly higher in all the ryes than in the wheat control (+37%), without any differences being observed among the rye genotypes in either location. Instead, there was no difference between rye and the wheat control as far as the calculated A/X ratio is concerned. The rye varieties had a higher β-glucan content than the wheat variety, that is, 1.2-fold greater at both sites. No significant differences among the rye varieties were found in the hilly site, whereas in the mountain site the highest value of β-glucans and the lowest were registered for Susa and for the commercial rye, respectively. The analysis of TDF was performed without any replicate, thus two-way ANOVA was not applied to this parameter. The rye varieties recorded higher values of TDF than the wheat control (on average +4% in the hilly site, and +5% in the mountain one), with the highest values observed for the hybrid rye in the hilly environment (16.7%) and for Susa in the mountain environment (16.9%).

The year had a significant effect on some of the analyzed parameters, and differences were detected between the two environments. TW was significantly higher in 2020 in the hilly site (+3%), whereas no significant differences between the two years were observed for the mountain site. The ash content was influenced to a great extent by the year in both environments, with higher values observed in 2021 than in 2020 in the hilly site (+9%), while an opposite trend was observed in the mountain site (+4% in plots grown during 2020 compared to 2021). Two-way ANOVA showed a significant effect of the year (*p* < 0.05) on both the A/X ratio and β-glucans in the mountain environment, with higher values observed in 2020 than in 2021 (+4 and +8%, respectively). The G × Y interaction was only significant for β-glucans in the mountain environment, with more marked differences among the rye genotypes being observed in 2020 (data not shown). In the first year, the commercial population cultivar presented a β-glucan content that was significantly lower than all the other rye genotypes (-20%), whereas this difference was not recorded in the second harvest.

### 3.5 Phenolic acids and antioxidant capacity of the whole-grain flour

The effect of genotype and year on the content and composition of the SPAs and CWBPAs of the whole-grain wheat and rye flours is shown in **Tables 3**, **4**, respectively. Two-way ANOVA showed a significant effect of the genotype on the total SPA content in the hilly and mountain environments, whereas the CWBPA content was not influenced by the genotype in either site. The SPA content was observed to be higher (+60%) in all the rye varieties than in the wheat control in both environments. Maira registered the greatest concentration of SPAs, followed by Susa, in the hilly site, while the highest SPA content in the mountain site was reached by both the Maira and Susa ryes, although Tanaro and the hybrid rye did not differ significantly from either. Sinapic acid was the most abundant SPA and was detected in all the analyzed genotypes, where it was found to represent 55-61% of the total SPAs, followed by ferulic acid (23-33%), *p*-coumaric and vanillic acid, which only accounted for 3-4% and 2-6% of the total share, respectively. A characteristic profile, which depended upon the considered genotype, was observed. Overall, the rye varieties presented a significantly higher concentration of sinapic, ferulic and *p*-coumaric acids in both sites than the wheat control, and significant differences were detected among the varieties and between the two growing environments. Conversely, the vanillic and syringic acid contents were significantly higher in the wheat variety.

**Table 3:** Effect of the genotype, the harvest year and their interaction on the total soluble phenolic acid content and on the content of the individual soluble compounds^a^ of the whole-grain flour of the investigated wheat and rye cultivars, cultivated in two different marginal Alpine environments.

Environment	Factors	Source of variation	SPAs (mg kg^-1^)		GA (mg kg^-1^)		PCA (mg kg^-1^)		HBA (mg kg^-1^)		VA (mg kg^-1^)		CA (mg kg^-1^)		SRA (mg kg^-1^)		*p*-CA (mg kg^-1^)		FA (mg kg^-1^)		SA (mg kg^-1^)	
Hilly (450 m)	**Genotype (G)**	Bread wheat	107	d	< LOQ		0.6	c	2.6	ab	6.3	a	0.3	a	6.2	a	3.4	c	22.9	e	65.0	c
		Susa rye	188	b	< LOQ		3.5	b	2.5	ab	5.6	ab	1.6	a	2.8	b	9.0	a	60.6	b	101.9	a
		Maira rye	202	a	< LOQ		4.8	a	3.0	a	4.9	b	2.1	a	2.2	b	9.5	a	69.6	a	105.6	a
		Tanaro rye	159	c	< LOQ		4.7	a	2.1	bc	5.0	b	0.6	a	2.4	b	8.5	a	46.9	c	89.0	b
		Commercial rye	160	c	< LOQ		3.3	b	2.3	bc	4.6	b	0.7	a	2.5	b	7.0	b	47.7	c	91.9	b
		Hybrid rye	148	c	< LOQ		3.9	ab	1.8	c	3.5	c	0.3	a	2.7	b	5.6	b	39.0	d	91.0	b
		*p* (F)	***		–		***		***		***		ns		***		***		***		***	
	**Year (Y)**	2020	187	a	< LOQ		4.4	a	2.6	a	6.1	a	0.8	a	3.4	a	8.6	a	57.2	a	103.6	a
		2021	132	b	< LOQ		2.3	b	2.1	b	3.8	b	1.2	a	2.9	b	5.5	b	37.8	b	76.3	b
		*p* (F)	***		–		***		***		***		ns		**		***		***		***	
	**G × Y**	*p* (F)	***		–		**		ns		ns		ns		***		***		***		**	
Mountain (900 m)	**Genotype (G)**	Bread wheat	81	c	0.3	a	1.4	d	2.1	a	4.8	a	0.1	b	4.6	a	2.1	b	20.1	c	45.1	c
		Susa rye	136	a	< LOQ	a	3.6	bc	1.7	bc	3.6	bc	1.0	a	1.9	bc	4.8	a	42.4	a	77.2	a
		Maira rye	134	a	0.4	a	3.0	c	2.0	ab	3.2	bc	0.8	a	1.4	c	4.4	a	41.5	ab	77.5	a
		Tanaro rye	131	ab	< LOQ	a	4.2	b	1.6	c	3.6	b	0.6	ab	2.0	bc	4.0	a	36.0	ab	78.5	a
		Commercial rye	110	b	< LOQ	a	3.0	bc	1.5	c	2.7	c	0.9	a	1.5	c	3.9	a	32.1	b	64.1	b
		Hybrid rye	129	ab	< LOQ	a	5.4	a	1.7	c	3.2	bc	1.1	a	2.5	b	3.8	a	32.4	b	79.3	a
		*p* (F)	***		ns		***		***		***		***		***		***		***		***	
	**Year (Y)**	2020	120	a	0.2	a	4.1	a	1.6	b	3.7	a	0.9	a	2.2	a	4.2	a	33.9	a	69.4	a
		2021	122	a	0.0	a	2.9	b	1.9	a	3.2	b	0.7	a	2.3	a	3.5	b	35.1	a	72.7	a
		*p* (F)	ns		ns		***		***		*		ns		ns		**		ns		ns	
	**G × Y**	*p* (F)	***		ns		***		***		**		ns		ns		ns		**		***	

a SPAs, soluble phenolic acids; GA, gallic acid; PCA, protocatechuic acid; HBA, hydroxybenzoic acid; VA, vanillic acid; CA, caffeic acid; SRA, syringic acid; p-CA, p-coumaric acid; FA, ferulic acid; SA, sinapic acid; LOQ, limit of quantification. The results are expressed on a DW basis. Means followed by different letters are significantly different, according to the REGW-F test [(*) *p* (F) ≤ 0.05, (**) *p* (F) ≤ 0.01, (***) *p* (F) ≤ 0.001, and ns, non-significant].

**Table 4:** Effect of the genotype, the harvest year and their interaction on the total cell wall-bound phenolic acid content and on the content of the individual insoluble compounds^a^ of the whole-grain flour of the investigated wheat and rye cultivars, cultivated in two different marginal Alpine environments.

Environment	Factors	Source of variation	CWBPAs (mg kg^-1^)		GA (mg kg^-1^)		PCA (mg kg^-1^)		HBA (mg kg^-1^)		VA (mg kg^-1^)		CA (mg kg^-1^)		SRA (mg kg^-1^)		*p*-CA (mg kg^-1^)		FA (mg kg^-1^)		SA (mg kg^-1^)	
Hilly (450 m)	**Genotype (G)**	Bread wheat	1004	a	1.3	a	1.0	c	3.6	a	6.2	a	9.8	a	7.7	a	36.9	c	820.8	a	116.7	b
		Susa rye	919	a	3.2	a	4.2	b	2.4	bc	4.8	bc	9.1	a	2.7	bc	50.5	b	696.2	bc	146.0	ab
		Maira rye	1052	a	2.3	a	3.3	bc	3.2	ab	5.2	b	4.8	a	2.9	bc	61.3	a	815.6	ab	150.7	ab
		Tanaro rye	962	a	1.7	a	7.4	a	1.5	d	3.9	c	8.1	a	1.8	c	53.5	ab	719.7	bc	164.5	a
		Commercial rye	959	a	2.8	a	2.0	bc	2.5	bc	4.7	bc	3.6	b	3.5	b	49.9	b	742.5	abc	147.2	ab
		Hybrid rye	917	a	2.4	a	3.9	bc	1.8	cd	4.6	bc	6.8	a	2.2	bc	40.3	c	695.6	c	159.2	ab
		*p* (F)	ns		ns		***		***		***		***		***		***		***		*	
	**Year (Y)**	2020	1176	a	3.2	a	4.4	a	2.8	a	6.3	a	8.8	a	4.2	a	58.6	a	919.9	a	167.1	a
		2021	469	b	1.4	b	2.9	b	2.2	b	3.5	b	6.3	b	2.8	b	38.6	b	582.7	b	128.6	b
		*p* (F)	***		**		*		***		***		***		***		***		***		***	
	**G × Y**	*p* (F)	***		ns		*		ns		*		**		**		***		***		**	
Mountain (900 m)	**Genotype (G)**	Bread wheat	998	a	2.0	a	2.8	a	3.5	a	5.6	a	11.7	a	4.0	a	42.2	bc	809.1	a	114.4	b
		Susa rye	974	a	1.2	a	4.3	a	2.1	b	4.2	b	10.2	ab	2.8	ab	48.6	abc	440.0	a	160.4	a
		Maira rye	885	a	2.3	a	3.0	a	2.6	b	4.1	b	9.2	ab	2.3	b	55.3	a	677.1	a	129.4	ab
		Tanaro rye	994	a	3.5	a	3.3	a	2.2	b	4.5	b	7.8	b	5.9	ab	50.0	ab	767.7	a	149.3	a
		Commercial rye	931	a	1.5	a	3.7	a	2.3	b	4.1	b	8.4	ab	2.5	b	49.7	ab	713.3	a	145.1	ab
		Hybrid rye	935	a	0.9	a	4.3	a	2.2	b	3.7	b	10.1	ab	2.3	b	39.7	c	718.0	a	153.4	a
		*p* (F)	ns		ns		ns		***		**		*		**		***		*		**	
	**Year (Y)**	2020	1047	a	2.6	a	5.1	a	2.4	a	5.0	a	10.5	a	3.3	a	56.3	a	811.0	a	150.5	a
		2021	859	b	1.2	b	2.0	b	2.5	a	3.8	b	8.6	b	4.3	a	38.9	b	664.1	b	133.5	b
		*p* (F)	***		**		***		ns		***		**		ns		***		***		*	
	**G × Y**	*p* (F)	ns		ns		**		ns		ns		ns		ns		ns		ns		ns	

a CWBPAs, cell wall-bound phenolic acids; GA, gallic acid; PCA, protocatechuic acid; HBA, hydroxybenzoic acid; VA, vanillic acid; CA, caffeic acid; SRA, syringic acid; p-CA, p-coumaric acid; FA, ferulic acid; SA, sinapic acid. The results are expressed on a DW basis. Means followed by different letters are significantly different, according to the REGW-F test [(*) *p* (F) ≤ 0.05, (**) *p* (F) ≤ 0.01, (***) *p* (F) ≤ 0.001, and ns, non-significant].

CWBPAs constituted most of the phenolic acid concentration, and on average represented 85-91% of the total phenolic acids. Ferulic acid was the predominant phenolic acid detected in the bound form, accounting for 76-81% of total bound phenolics. The other abundant CWBPAs were: sinapic acid (12-17%) and *p*-coumaric acid (4-6%). Gallic acid showed the lowest values, ranging from 0.9 to 3.5 mg kg^-1^ in the bound form, but it was only detected in traces in the soluble form. The genotype did not influence the total content of CWBPAs, although the effect was significant for most of the individual compounds. The wheat variety registered the highest ferulic acid value in the hilly site, although Maira and the commercial rye did not differ significantly from the control. As far as sinapic acid is concerned, the rye varieties showed higher values than the control, although, in some cases, the differences were not significant. As previously observed for the SPAs, the concentrations of bound vanillic and syringic acids were higher in the wheat variety than in the ryes, but they only accounted for a small share of the total content of the bound phenolic acids (0.2-0.7%). Additionally, the variation patterns in the phenolic acid compositions differed among the rye genotypes.

Apart from the genotype, the year factor also affected the SPA and CWBPA concentrations. The highest content was observed in 2020 in both sites and for both parameters, but not for the SPAs in the mountain site, whose content did not differ significantly over the two years. The 2020 harvest was in fact characterized by higher SPA and CWBPA contents in the hilly environment (+42 and +53%, respectively), while the CWBPAs increased by about 22% in the mountain environment in 2020, compared to 2021. A similar trend was observed for the individual phenolic acids, although some of them were not influenced by the year.

The G × Y effect was significant for both SPAs and CWBPAs in the hilly site, with marked differences among the rye genotypes being observed in 2020. Maira had a significantly higher content of both forms of phenolic acids in the 2020 harvest, while the differences were less distinct (SPAs) or were not detected (CWBPAs) among the genotypes in 2021 (data not shown). The interaction was only significant for SPAs in the mountain environment, with more marked differences among the rye genotypes being observed in 2021, with Maira presenting a higher content than all the other genotypes (data not shown).

**Figure 2:**
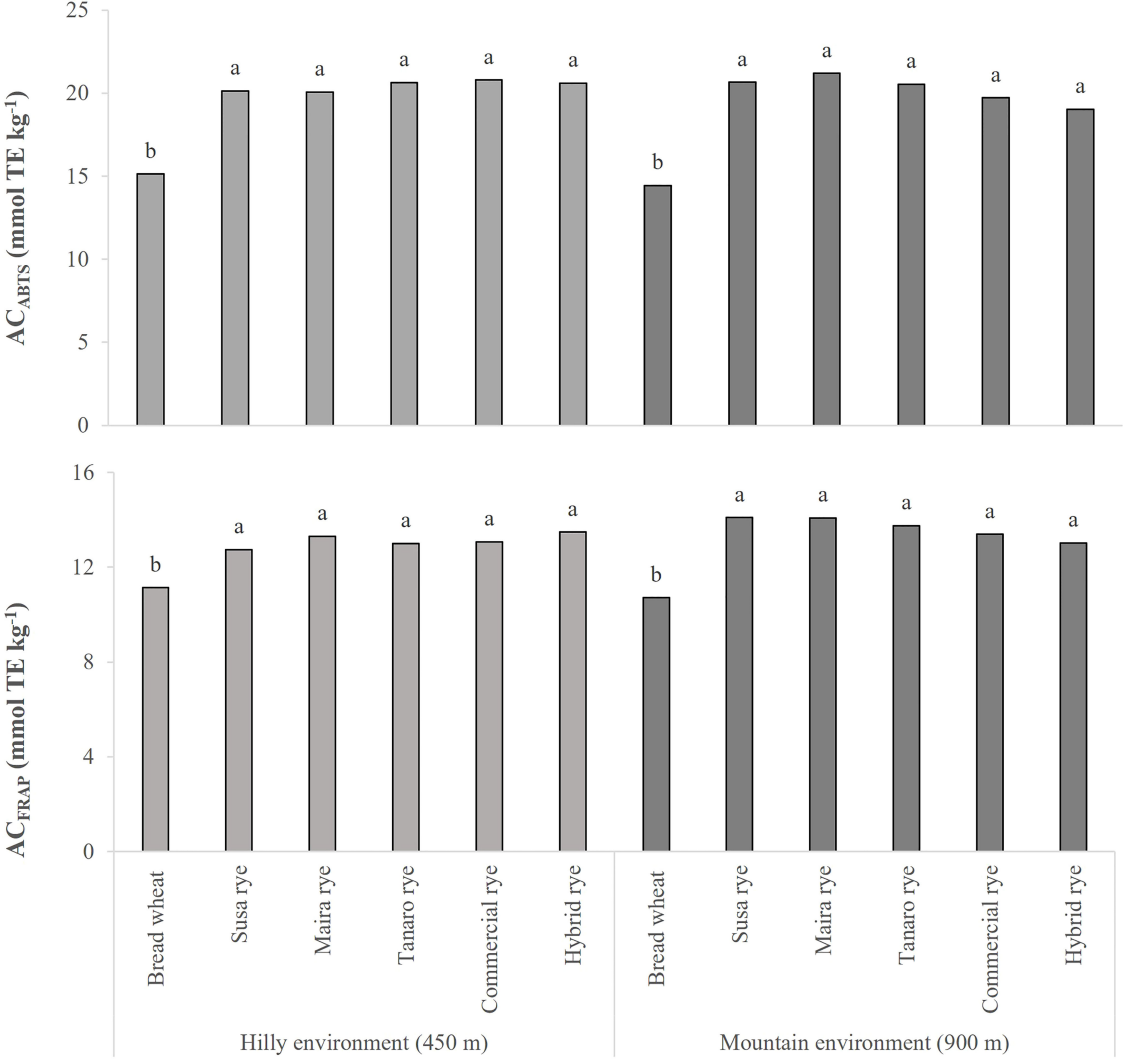
Comparison of the antioxidant capacity (AC) of the wheat and rye whole-grain flours, measured by means of two different methods (ABTS and FRAP assays). The data are averaged over 2 years and for 3 replications. The results are expressed on a DW basis. Different letters on bars indicate significant differences [*p* (F) ≤ 0.05].

The AC of the wheat and rye whole-grain flours was measured by means of two different assays, ABTS and FRAP. As shown in **Figure 2**, the AC values of the rye varieties were significantly higher than in the wheat control in both sites, and this result was confirmed by both assays. Furthermore, no significant differences were found between the five examined rye genotypes. In the hilly site, the AC of the rye varieties was on average 35 and 18% higher than in the wheat control, while the increase was recorded to be about 40 and 28% (ABTS and FRAP assays, respectively) in the mountain site. Even though the AC of the rye landraces did not differ significantly from those of the commercial and the hybrid ryes, they were characterized by higher AC values in the mountain site (on average +7%). The growing year only had a significant effect on the AC_ABTS_ of the plots cultivated in the hilly environment (data not shown), where it resulted in higher levels in 2020 (+10%) than in 2021, in accordance with the higher phenolic acid concentration.

Significant relationships were found between the phytochemicals and AC of the analyzed whole-grain flours (**Table S4**). Significant correlations were observed between the AC, measured with both assays, and the SPAs (r = 0.507 and r = 0.361, according to the ABTS and FRAP assays, respectively), whereas no correlation was observed between the AC and CWBPAs. The three most abundant phenolic acids, that is, sinapic, ferulic and *p*-coumaric, were highly correlated to AC in the soluble form, with the exception of *p*-coumaric acid and AC_FRAP,_ whose correlations was not significant (data not shown). Less marked, but still significant correlations were registered for sinapic and *p*-coumaric acids in the insoluble form and AC (data not shown).

### 3.6 Rheological parameters of the flour blends

Twelve blends of refined flour were formulated for bread making, with a 40% replacement of the whole-grain flour obtained from the wheat and rye genotypes cultivated in the hilly and mountain environments, and characterized for their rheological properties; the refined commercial wheat flour (no replacement) was considered as the control.

The refined flour control in the Mixolab test required a water supply of 55.8% (14% basis) to reach an adequate dough consistency (data not shown). The replacement with whole-grain wheat flour increased the amount of water (WA) required for the hydration process of the flour to 56.9% (average of the two sites), while the inclusion of whole-grain rye flour led to a further increase in the WA (59.0%, average of the two sites). The highest WA values were observed for the Tanaro blend in the hilly site and for the Maira blend in the mountain site (59.5%). The functionality of the whole-grain wheat and rye blends was estimated by determining the SRC profile and by comparing it with the SRC profile of the refined control (data not shown). SRC tests were carried out using two different solvents, namely water (W-SRC) and sucrose solutions (S-SRC), to provide information on some of the chemical and physical properties of the samples, and particularly on the ability of specific constituents to absorb and retain a given solvent. The water holding capacity (W-SRC) was similar for the refined control and the blend with whole-grain wheat flour substitution (76.5 and 75.3%, respectively, data averaged over two sites), while increasing values were observed for the rye mixes (+24% in both sites, compared to the refined flour). Maira registered the highest W-SRC value in both the hilly and the mountain environments (100.1 and 98.3%, respectively), while the lowest value was recorded for the commercial rye (90.8%). The rye blends were characterized by higher S-SRC values than the refined control (about +7% and +13% in the hilly and mountain sites, respectively).

Increasing DDT values were observed, for the adapted hydration protocol, for the whole-grain wheat and rye blends (9 min), compared to the refined flour (3 min), while DS progressively decreased (data not shown), thus confirming the role of increasing levels of dietary fiber on the rheological behavior of the whole-grain blends. However, a similar behavior was recorded for all the rye blends when the standard protocol was adopted. On the other hand, significant differences were found among the rye genotypes using the FAST protocol at constant WA, as a result of the higher mechanical shear stress (**Table 5**). The DDT parameter was similar for the refined control and the whole-grain wheat flour substitution, whereas it decreased by about 1 min for the mixes containing whole-grain rye flour in both sites. The DDT of the rye blends were homogeneous, with a few exceptions (the commercial and the hybrid rye showed lower DDT values in the hilly and the mountain sites, respectively). On the other hand, the inclusion of whole-grain wheat negatively affected the DS, which was significantly lower than the refined control (-38% in both sites). A further decrease in DS was registered for the inclusion of whole-grain rye (-50 and -54% in the hilly and mountain environments, respectively), although to a different extent, depending on the rye variety. C1, the maximum torque, was significantly higher for the rye varieties than for the refined control and the whole-grain wheat blend. Significant differences were also detected for the (C1-C2) parameter (thermal weakening) in both sites. Maira showed a significantly high value, in the hilly site in particular, which was similar to the refined control and the whole-grain wheat blend, whereas the other rye blends presented lower values, albeit to different extents. The samples from the mountain site were more homogenous, except for the Susa blend. Gelatinization (C3-C2) was significantly higher for the whole-grain wheat blend than for the rye blends in both sites. Among the rye varieties, Tanaro was the one that was characterized by the highest gelatinization, while Maira and the hybrid rye were characterized by the lowest. The Maira blend was characterized by the highest cooking stability range (C3-C4), which, overall, was higher for all the rye blends than for the wheat substitution and the refined control, and it was also characterized by the lowest cooling setback (C5-C4).

**Table 5:** Effect of the bread formulation, the harvest year and their interaction on the rheological parameters^a^ of a refined wheat flour (used as a control) and twelve blends containing refined wheat flour and wheat or rye whole-grain flour (WGF) at a 40% substitution level.

Environment	Factors	Source of variation	DDT (min)		DS (min)		C1 (Nm)		C1-C2 (Nm)		C3-C2 (Nm)		C3-C4 (Nm)		C5-C4 (Nm)	
Hilly (450 m)	**Bread formulation (BF)**	Refined control	3.4	a	4.3	a	1.42	b	0.50	ab	1.84	e	0.15	c	0.74	c
		40% WGF bread wheat	3.4	a	2.7	b	1.35	c	0.51	ab	2.16	a	0.23	c	0.98	b
		40% WGF Susa rye	2.7	b	2.4	c	1.53	a	0.45	de	2.00	c	0.51	b	1.10	a
		40% WGF Maira rye	2.6	bc	2.1	d	1.44	b	0.53	a	1.84	e	1.00	a	0.62	d
		40% WGF Tanaro rye	2.7	bc	2.1	cd	1.54	a	0.47	cd	2.08	b	0.51	b	1.03	ab
		40% WGF commercial rye	2.6	c	2.0	d	1.54	a	0.50	bc	2.01	c	0.54	b	0.96	b
		40% WGF hybrid rye	2.7	b	2.3	cd	1.46	b	0.43	e	1.97	d	0.49	b	0.98	b
		*p* (F)	***		***		***		***		***		***		***	
	**Year (Y)**	2020	2.9	a	2.5	a	1.4	b	0.45	b	1.92	b	0.55	a	0.83	b
		2021	2.8	a	2.5	a	1.5	a	0.52	a	2.05	a	0.43	b	1.00	a
		*p* (F)	ns		ns		***		***		***		***		***	
	**BF × Y**	*p* (F)	***		ns		***		***		***		***		***	
Mountain (900 m)	**Bread formulation (BF)**	Refined control	3.4	a	4.3	a	1.42	c	0.50	a	1.84	f	0.15	e	0.74	c
		40% WGF bread wheat	3.5	a	2.7	b	1.39	c	0.51	a	2.11	a	0.23	d	1.01	a
		40% WGF Susa rye	2.7	b	2.1	c	1.48	b	0.46	b	1.96	cd	0.60	c	0.94	ab
		40% WGF Maira rye	2.5	bc	2.1	cd	1.50	ab	0.51	a	1.93	de	0.75	a	0.85	b
		40% WGF Tanaro rye	2.6	bc	2.1	cd	1.54	a	0.52	a	2.00	b	0.67	b	0.95	ab
		40% WGF commercial rye	2.5	bc	1.9	de	1.51	ab	0.52	a	1.96	c	0.67	b	0.91	ab
		40% WGF hybrid rye	2.4	c	1.8	e	1.51	ab	0.52	a	1.91	e	0.59	c	0.89	ab
		*p* (F)	***		***		***		***		***		***		***	
	**Year (Y)**	2020	2.8	a	2.4	a	1.4	b	0.50	a	1.91	b	0.65	a	0.81	b
		2021	2.8	a	2.4	a	1.5	a	0.51	a	2.01	a	0.39	b	0.99	a
		*p* (F)	ns		ns		***		ns		***		***		***	
	**BF × Y**	*p* (F)	***		***		***		***		***		***		***	

^a^DDT, dough development time; DS, dough stability; C1, peak of 1.1 Nm ± 0.05 at 30 °C; C1-C2, thermal weakening; C3-C2, gelatinization; C3-C4, cooking stability; C5-C4, cooling setback. The results are expressed on a DW basis. Means followed by different letters are significantly different, according to the REGW-F test [(*) *p* (F) ≤ 0.05, (**) *p* (F) ≤ 0.01, (***) *p* (F) ≤ 0.001, and ns, non-significant].

The source genotypes of WGF were cultivated in two different marginal Alpine environments. The considered rheological parameters were obtained by applying the Mixolab^®^ FAST protocol, using 56% of constant WA.

### 3.7 Bread characterization

Fourteen breads (one refined flour used as a control, one bread wheat variety, five rye varieties for each site) were produced with the previously described flours and were analyzed to establish some technological and antioxidant properties.

One-way ANOVA showed a significant effect of the bread formulation on the bread volume, with differences emerging between the two growing locations (**Table 6**). As far as the breads produced with flour obtained from the hilly site are concerned, a decrease in the volume was already observed in the bread produced with the whole-grain wheat flour replacement (-5%). Breads made with rye flour showed a further reduction (-22% on average), with differences emerging among the varieties. The highest value was observed for the bread made with the Maira blend. As far as the mountain site is concerned, the volume of the whole-grain wheat bread was statistically similar to that of the refined control, while the rye breads presented significantly lower volumes (-25% on average), although these values were slightly more homogenous than those of the hilly site.

**Table 6:** Effect of the bread formulation on the bread-making aptitude^a^ of a refined wheat flour (used as a control) and twelve blends containing refined wheat flour and wheat or rye whole-grain flour (WGF) at a 40% substitution level.

Environment	Bread formulation	Bread volume (mL)		Color: lightness				Color: redness				Color: yellowness				Color: Δ*E*			
				Crust		Crumb		Crust		Crumb		Crust		Crumb		Crust		Crumb	
Hilly (450 m)	Refined control	1573	a	68.0	a	67.1	a	8.2	b	1.1	c	28.3	a	14.3	d	–		–	
	40% WGF bread wheat	1495	b	56.1	b	53.1	d	11.6	a	4.0	b	26.8	abc	16.0	c	8.2	b	16.2	a
	40% WGF Susa rye	1197	d	49.9	c	55.8	cd	14.2	a	4.4	ab	27.6	ab	17.4	abc	14.2	a	13.9	ab
	40% WGF Maira rye	1315	c	45.7	c	58.4	bc	13.6	a	5.1	a	24.3	c	18.8	a	18.9	a	12.1	bc
	40% WGF Tanaro rye	1280	c	49.2	c	60.4	b	13.3	a	4.6	ab	25.2	bc	18.6	a	15.3	a	10.2	c
	40% WGF commercial rye	1197	d	46.0	c	54.9	cd	14.2	a	4.3	ab	26.2	abc	16.8	bc	18.2	a	14.7	a
	40% WGF hybrid rye	1146	d	48.3	c	60.5	b	14.0	a	4.2	ab	27.1	ab	17.8	ab	15.7	a	9.7	c
	*p* (F)	***		***		***		***		***		**		***		***		***	
Mountain (900 m)	Refined control	1573	a	68.0	a	67.1	a	8.2	c	1.1	d	28.3	a	14.3	c	–		–	
	40% WGF bread wheat	1514	a	54.3	b	55.2	b	13.5	ab	3.4	c	27.9	b	13.8	c	9.7	b	14.0	a
	40% WGF Susa rye	1170	bc	45.9	c	57.0	b	13.7	ab	4.2	bc	25.2	c	16.8	ab	18.4	a	12.6	a
	40% WGF Maira rye	1139	c	47.4	c	57.6	b	13.9	ab	5.2	a	26.0	bc	18.3	a	16.9	a	12.7	a
	40% WGF Tanaro rye	1149	bc	48.4	c	58.0	b	14.9	a	4.4	ab	27.7	ab	18.8	a	15.7	a	12.3	a
	40% WGF commercial rye	1240	b	48.0	c	56.2	b	13.4	ab	4.7	ab	25.8	bc	17.8	ab	16.2	a	13.8	a
	40% WGF hybrid rye	1197	bc	54.2	b	56.4	b	12.1	b	3.9	bc	26.4	abc	16.3	b	10.1	b	13.1	a
	*p* (F)	***		***		***		***		***		***		***		***		ns	

a ΔE: the total color difference from the refined control. The results are expressed on a DW basis. Means followed by different letters are significantly different, according to the REGW-F test [(*) *p* "[(*) *p* (F) ≥ 0.05, (**) *p* (F) ≥ 0.01, (***) *p* (F) ≥ 0.001, and ns, non-significant].

The source genotypes of WGF were cultivated in two different marginal Alpine environments during 2019-20 growing season.

The color of the crust and crumbs of each bread was analyzed, and the Δ*E* parameter was assessed considering the refined bread as the control (**Table 6**). The replacement with whole-grain flour resulted in a reduction in the lightness (L*) value and an increase in the redness (a*) value of the crust, while the blue-yellow component (b*) was similar to that of the refined control, with the exception of Maira and Tanaro in the hilly site, while the values in the mountain site were more heterogeneous. Rye inclusion also affected the color of the crumbs, with differences among the varieties and between the environments. The rye breads from the hilly site appeared redder and yellower than the wheat bread and the refined control, but the lightness component was higher than that of the wheat bread and lower than that of the refined control. A similar trend was observed for the mountain site, but no significant differences were detected among the rye and wheat breads for the lightness component, which presented lower values than the refined control.

**Figure 3:**
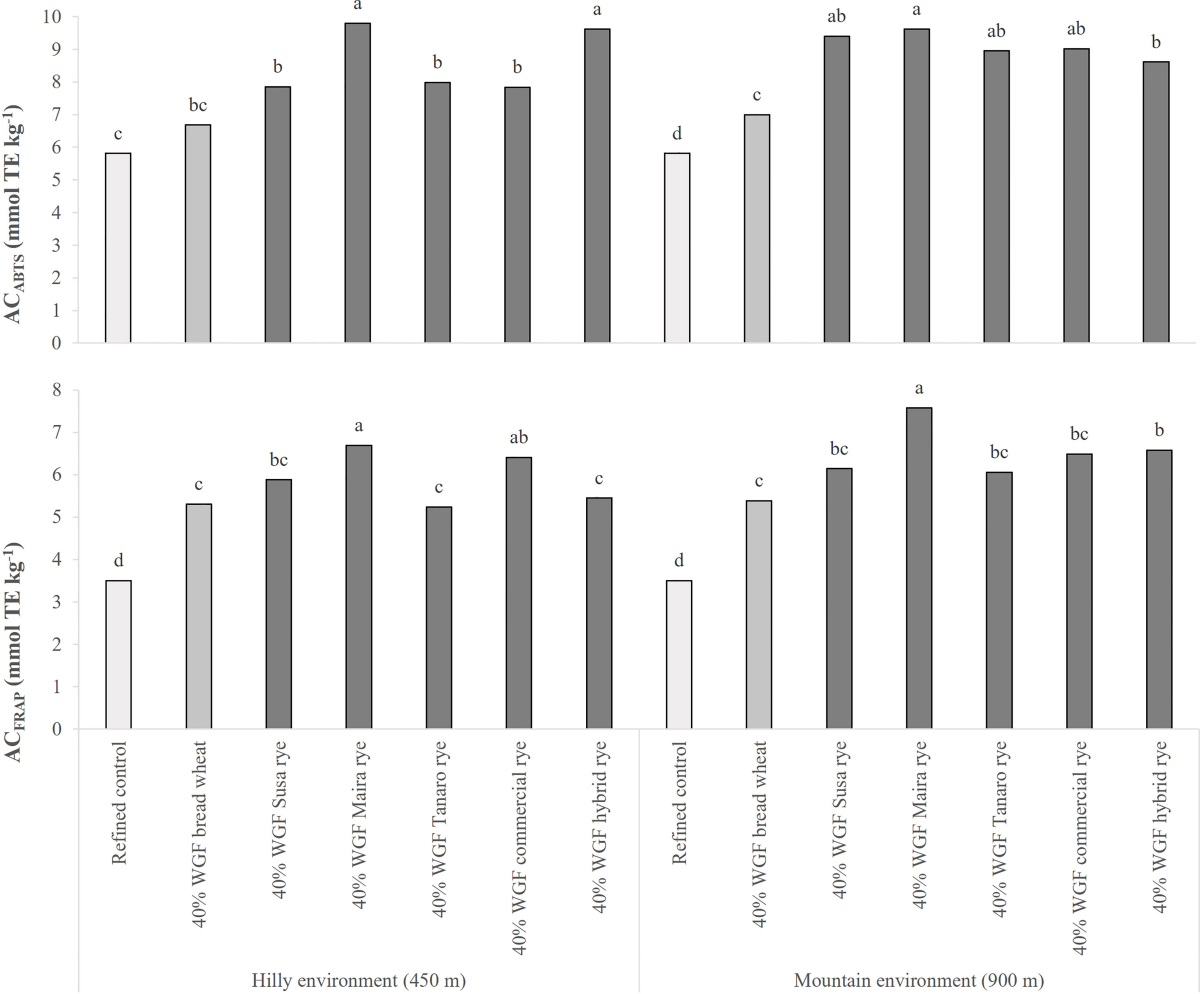
Comparison of the antioxidant capacity (AC) of the bread made of refined wheat flour (used as a control) and breads made of refined wheat flour and wheat or rye whole-grain flour (WGF) at a 40% substitution level, measured by means of two different methods (ABTS and FRAP assays). The source genotypes of WGF were cultivated in two different marginal Alpine environments during 2019-20 growing season. The data are the average of 3 replications. The results are expressed on a DW basis. Different letters on bars indicate significant differences [*p* (F) ≤ 0.05].

The AC of the breads increased significantly when the refined flour was replaced with whole-grain flour (**Figure 3**). A significant increase was recorded for the bread with whole-grain wheat, compared to the control, in both sites and for both assays, although the AC_ABTS_ value for the hilly site did not differ significantly from that of the control. The AC of the breads made with rye flour was significantly higher than the refined control, but similar or higher than the bread with whole-grain wheat flour. Large variations were observed among the rye varieties, contrary to what was observed for the whole-grain flours. The maximum observed increase was recorded for Maira, when employing both the ABTS (+69 and +65% in the hilly and mountain sites, respectively, compared to the refined control), and the FRAP (+91 and +116%) assays.

## 4 Discussion

The field experiments carried out in the present work offer evidence of the agronomical and qualitative differences between rye and bread wheat cultivated in marginal growing areas, such as in the Alpine valleys of Northwest Italy. These differences have already been pointed out for other growing areas, and in particular in northern, central and eastern Europe countries (Aprodu and Banu, 2017; Laidig et al., 2017; Johansson et al., 2021). No differences in yield potential were found between rye and wheat when comparing hilly and mountain environments. Indeed, although rye was found to be characterized by a lower TKW than wheat, this was compensated for with a greater number of kernels per ear. Rye was also characterized by higher plant heights and straw yields as, in the past, it was also grown to provide material for roofs and baskets, other than for flour (Gaetano et al., 2012). The rye kernels showed a lower TW and GPC than bread wheat, but presented, in both environments, higher concentrations of minerals, soluble fibers, and the soluble fraction of phenolic acids, which resulted in a superior AC of both the flours and the final baked products. Other studies have already pointed out the high AC of rye grains, due to their high content of compounds which have been shown to possess strong bioactivity *in vitro* (Bondia-Pons et al., 2009; Andersson et al., 2014). Phenolic compounds are regarded as potent antioxidants and have been suggested to play a role in the prevention of many degenerative diseases, mainly due to their antioxidative, anti-inflammatory and anti-carcinogenic effects (Li et al., 2008). The greater content of phenolic acids in the rye samples than in the wheat control should be ascribed to a higher contribution of the soluble fraction to the total amount. This hypothesis is in agreement with prior works (Nyström et al., 2008) and should be considered of great interest, as the non-bound fraction of phenolic acids is believed to be readily available for absorption in the small and large intestines of humans (Manach et al., 2004) and it has been indicated to be the most flavor-active, thereby contributing to the perceived flavor of rye products (Heiniö et al., 2008). Aprodu and Banu (2016), by means of the DPPH (2,2-diphenyl-1-picrylhydrazyl-hydrate) free radical scavenging method, observed a higher AC for rye than for wheat and triticale samples (1.4 and 1.3-times higher, respectively), while Michalska et al. (2007), who compared whole-grain rye breads with commercial wheat rolls, reported superior antioxidant properties for the rye baked samples.

As far as the bread-making properties are concerned, the inclusion of whole-grain rye in a bread formulation resulted in a higher WA and a lower DS than those of wheat, thus leading to reduced loaf volumes and darker baked products. A previous study investigated the influences of rye inclusion on dough behavior during leavening and on bread-related features (Colombo et al., 2022). The authors observed a positive effect of rye inclusion on dough development, as the dough rose faster and higher when rye was added to wheat. Furthermore, the bread features (volume, crumb firmness and crumb moisture) were similar to those of the control. These different outcomes can be ascribed to the different levels of rye inclusion (30% vs the 40% of the present study) and to the different protein contents of the rye flour. GPC is an important indicator of the bread-making aptitude of a flour, and it is usually correlated with the DDT of the dough and with the volume of the final bread. Nevertheless, no gluten network forms in rye flour during the dough-making step. Rye storage proteins are not able to form a strong viscoelastic network with good gas-retaining properties, as wheat gluten proteins are instead capable of forming, due to different composition and lower content (Meeus et al., 2020). The lack of the formation of gluten in a rye dough makes the swelling substances highly important for the dough and bread crumb structure. The carbohydrate fraction and the amylase activity were observed to play major roles in influencing the dough quality and baking performances of rye flour. Among the non-starch polysaccharides, pentosans, mainly arabinoxylans, are the main contributors to the high viscosity of rye dough, due to their high water-holding capacity (Deleu et al., 2020), which, in particular, characterizes the water-extractable fraction (WEAXs). The positive effect of WEAXs on bread quality has been related to an increased viscosity and elasticity of the dough and to an improved stabilization of the liquid films around the gas cells and, consequently, of the foam structure, thus resulting in improved gas retention and in an increased loaf volume and crumb softness (Buksa et al., 2013). Furthermore, arabinoxylans are the most abundant fibers in rye, ranging from 6.5 to 12.2% of DW, with 1.5-4% being WEAXs (Vinkx and Delcour, 1996; Nilsson et al., 1997; Hansen et al., 2003), and therefore contribute to a great extent to the beneficial effects related to gut health, bowel movement and increased satiety that are well-known for high-fiber cereal foods (Jonsson et al., 2018). In the present study, the total arabinoxylan contents of the rye varieties, which ranged between 8.4 and 8.9% (DW), were significantly higher than those of the wheat variety, as was the β-glucan component (1.9-2.2% DW). Furthermore, as far as bread making is concerned, the overall higher level of TDF that characterized the whole-grain rye flour resulted in higher SRC values, both S-SRC, which is associated with pentosan contents, and W-SRC, which provides information on all the compounds that are able to interact with water and which are characterized by a good water holding capacity (Aprodu and Banu, 2017). The competition of fibers with proteins for water resulted in rye doughs that were not sufficiently hydrated at 56% of WA and required shorter times to reach the maximum C1 torque, thereby leading to lower DDT and DS values than those of the refined wheat control. Indeed, the DDT and DS parameters were found to be negatively correlated with both TAX and the β-glucan content. The different dough resistances to mechanical stress, due to a lack of adequate hydration, between the wheat and rye doughs were confirmed by the higher C1 values of the rye doughs, which were hence characterized by insufficient plasticity and were difficult to process. A positive effect of the TAX and β-glucan component was registered for the cooking stability range (C3-C4), as the doughs containing rye developed a more stable starch gel under a heating constraint and were characterized by a higher amylase activity than the doughs of the whole-grain wheat and the refined control.

The differences between the rye genotypes and the wheat control were slightly greater in the hilly environment than in the mountain one, but only concerning a few agronomical and sanitary parameters, e.g. TKW, straw yield, HI and ergot sclerotia. On the other hand, the different behaviors observed between rye and wheat were similar for all the qualitative and phytochemical traits, and for the bread-making aptitude, in both of the considered marginal growing areas.

This paper also highlights the differences that exist in the qualitative traits between rye genotypes, when comparing commercial cultivars (one population and one hybrid) with local landraces, which were selected according to their genetic diversity, and were cultivated side by side in the same marginal environments. Overall, only very small differences were reported for the agronomical and yield traits. The higher potential yield of the hybrid cultivar is the most evident trait, even in marginal environments, and it benefits from the heterosis effect, which resulted in a greater plant functionality and stability and in a higher HI. The superior yield capacity of hybrids than that of population cultivars has already been reported for intensive cropping systems (Hansen et al., 2004; Miedaner and Hübner, 2011). However, the hybrid cultivar was also the most susceptible to sanitary risks, and for the occurrence of ergot sclerotia in particular, and thus potentially alkaloids that are able to cause severe mycotoxicosis in both humans and animals, with an always higher content than the maximum regulatory levels (EU No. 2021/1399). Recent experiments have observed a much higher degree of ergot severity in hybrid cultivars than in open-pollinated population cultivars (7.5 vs 2.2%, on average), due to the occurrence, in the former, of poorly restored plants that shed less pollen (Mirdita et al., 2008; Mirdita and Miedaner, 2009). As ergot is able to efficiently infect florets that have not yet pollinated or those that have just pollinated, even a reduced amount of pollen favors ergot infection. This occurs when a hybrid cultivar is not fully restored to fertility and therefore, on average, sheds less pollen than population cultivars or landraces, which are instead characterized by full pollen shedding (Miedaner and Geiger, 2015). The risk related to this disease was found to be higher in the hilly environment than in the mountain one, and in particular for rainy spring seasons during the flowering period. On the other hand, the risk of contamination of other mycotoxins, such as DON, was observed to be very low in the considered marginal environments, and similar to what has been reported for other Alpine areas (Colombo et al., 2022). A previous study, carried out in an Alpine valley in Northeast Italy, investigated the differences between modern rye cultivars and South Tyrolean landraces collected from local farmers (Peratoner et al., 2016). The authors observed that traits related to grain yield and plant stability were significantly less favorable for landraces than for modern cultivars, as were the investigated parameters related to baking quality. On the other hand, they found that landraces showed a good adaptation to the altitude of the site of origin. In the present study, no significant differences were detected among the landraces and commercial rye cultivars for the proximate and dietary fiber composition. Conversely, Hansen et al. (2003) found higher TDF and TAX contents in population varieties than in hybrid cultivars, while the hybrid rye considered in the present work was characterized by higher TDF contents in the hilly site and retained a high content in the mountain site.

The rye landraces were characterized by higher levels of SPAs and CWBPAS, in both environments, than the conventional rye cultivars, although the AC of the whole-grain flour, as measured by means of two assays, was similar among the rye genotypes, in agreement with that of Leoni et al. (2021) for the whole-grain flour of rye landraces from a Northwest Italian valley. Instead, Kulichová et al. (2019) reported considerable differences in the antioxidant properties of 19 rye genotypes, as measured by four different methods. The authors ascribed the different profiles and amounts of antioxidants in the rye genotypes to the provenience of the genotypes from different parts of Europe, and to their different morphological and agronomical traits. A larger variation has been recorded in the present study for the AC of bread obtained from the rye varieties than for the AC of the source flours, with landraces on average being characterized by higher antioxidant properties than the conventional varieties. Many studies have reported that the antioxidant status of flour can be changed by the bread-making process. Changes in the antioxidant activity of bread can in particular occur because the baking step leads to the destruction of natural-labile antioxidants and to the formation of other thermally-induced compounds that have antioxidant properties, with differences that depend upon the chemical content of the flour and the technological conditions adopted during the process (Delgado-Andrade et al., 2010).

The differences among the rye genotypes were more evident for distinctive agronomic and qualitative parameters, when specific landraces were considered. In particular, the genotype from the Maira Valley showed the most notable morphological differences, as it had the tallest and thinnest culm and suffered from lodging phenomena, which reduced its yield potential and HI. Maira also showed the main differences as far as the qualitative traits are concerned, compared to the other rye genotypes. The proximate composition analyses revealed the superiority of Maira over the rye landraces and commercial cultivars for bread-making purposes, as it was characterized by the highest protein and ash contents in both environments. On the other hand, the Susa and Tanaro landraces were characterized by low GPC values, like those of the hybrid rye, but higher TAX and β-glucan contents. Maira showed the highest SPA and CWBPA contents in the hilly site, while Susa registered the highest values in the mountain site. The dough formulation including the Maira flour showed the greatest ability to absorb and retain a highly concentrated sugar solution in both locations, and this was followed by Susa, which was in turn characterized by the highest level of soluble fibers. Differences in protein network weakening among the rye varieties were registered at constant hydration. In the hilly site, the blend including Maira was characterized by the highest thermal weakening (C1-C2), which can be explained by the high GPC of the source flour, which in turn resulted in bread characterized by the highest volume for that location. On the other hand, the blend containing the hybrid rye cultivated in the hilly site had a considerably low (C1-C2) value, mainly due to the low GPC and high TDF contents observed in the same site, which led to a final bread characterized by the lowest volume. Low bread volumes were registered for the blend with Susa in both environments, which was characterized by high β-glucan, high TDF and low GPC contents. Furthermore, this blend presented a low C2 value for the adapted hydration (data not shown), thus suggesting that the dough resisted deformation during kneading and heating, and this was confirmed by the DDT value, which was higher than those of all the other analyzed blends. The rye mixes presented a similar or lower starch gelatinization level (C3-C2) than the refined control, due to starch dilution of the DF compounds, except for the Maira blend, which registered much lower values and was also characterized by a low cooling setback (C5-C4). Furthermore, the Maira-based bread retained the highest AC, as measured by both assays, in both sites, thus suggesting that this landrace, in combination with wheat flour, was the most suitable for bread making, as it showed the best performances when the rheological and antioxidant properties were considered.

**Figure 4:**
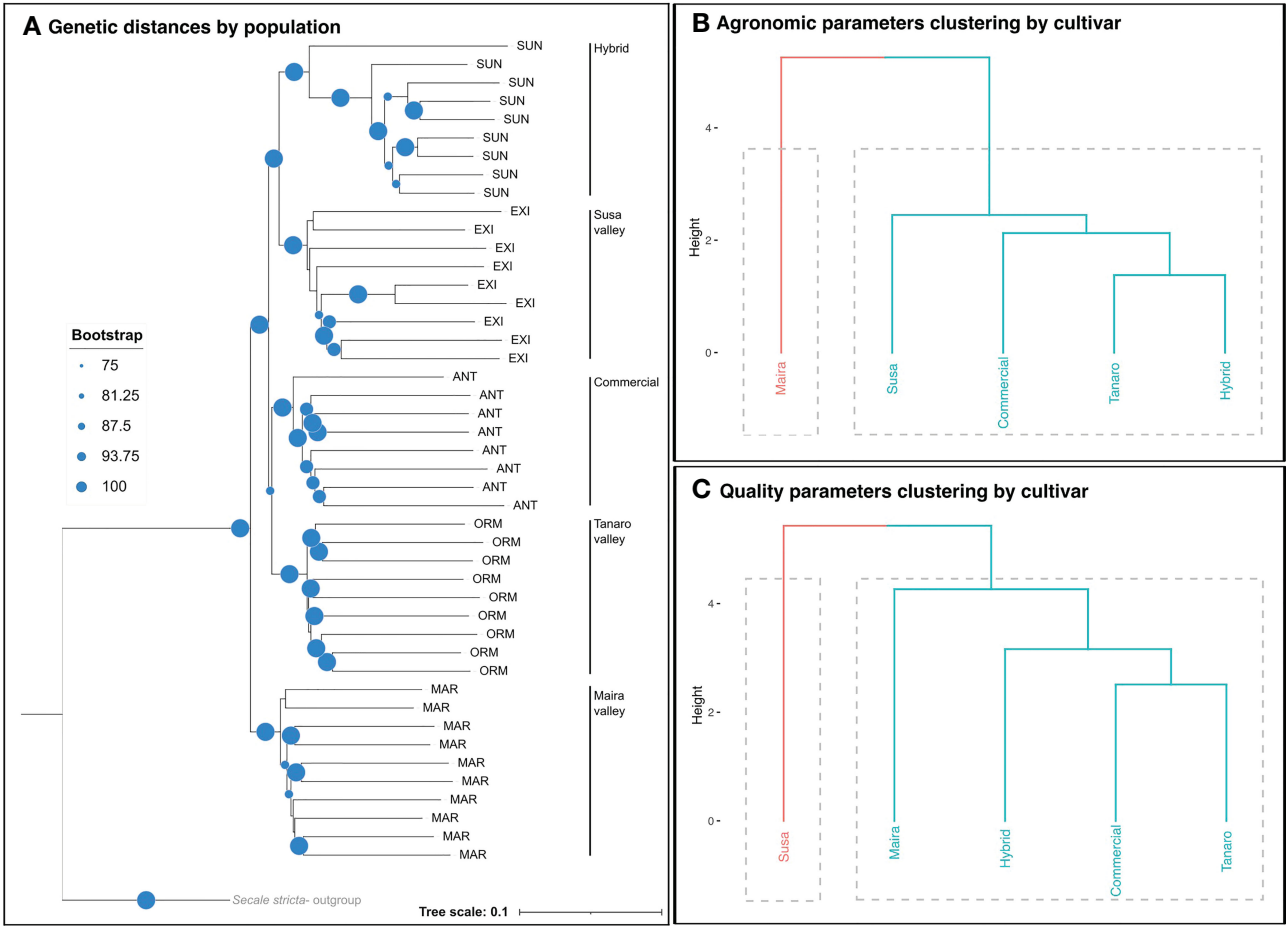
Genetic distances between the rye genotypes^a^ visualized by means of a neighbor joining tree **(A)**; hierarchical clustering of the rye genotypes based on agronomic **(B)** and qualitative parameters **(C)**. ^a^SUN, EXI, ANT, ORM, MAR refer to the accession codes adopted in Adamo et al. (2021).

Our results highlighted that genetically diverse genotypes have distinctive agronomic features which have been observed in different cultivation environments. Individuals from different cultivars resulted to be separated into coherent clades, thus revealing that the rye genotypes were well differentiated among populations, and the clades were well supported, with bootstrap values ≥ 75 (**Figure 4A**). This genetic distinctiveness can be ascribed to the geographical isolation and historical background of rye, which was cultivated as a staple subsistence crop in many isolated valleys in the Northwest Italian Alps (Adamo et al., 2021). However, the Tanaro and Susa ryes were positioned close to the commercial population cultivar and to the hybrid cultivar, respectively (**Figure 4A**), thus possibly indicating a lower genetic isolation for the former, and a less distinct origin for the second, with respect to the Maira rye. In fact, as also pointed out in Adamo et al. (2021), many local rye varieties were shown to share a common genetic background with the hybrid and the commercial ryes. Overall, the genetic distance from conventional rye cultivars was found to influence specific agronomic and qualitative traits, even in the marginal Alpine areas, with differences being observed among the individual landraces. Hierarchical clustering of the agronomic parameters indicated that Maira was part of a separated cluster, that showed a high genetic distance from all the other rye cultivars (**Figure 4B**). On the other hand, from the qualitative perspective, Susa was found to be part of a cluster that was separated from the other rye cultivars, and this was followed by Maira (**Figure 4C**).

In summary, to the best of the authors’ knowledge, this study is the first to have investigated a wide multiplicity of agronomic, nutritional, and qualitative parameters of locally selected rye landraces grown in marginal environments, along with wheat and commercial rye cultivars. The genotype selected from Tanaro Valley was found to be close to the commercial population cultivar, from the genetic, agronomic, and qualitative points of view. Maira, according to the genetic investigation, was found to be qualitatively divergent from all the other analyzed commercial and landrace rye genotypes, and to have less favorable morphologic and agronomic traits. Nevertheless, it was also characterized by the highest content of health-related substances and a better bread-making aptitude. A good bioactive compound content was also registered for Susa, together with a relevant number of soluble fibers, which, however, proved to be detrimental for its rheological properties. The present work has demonstrated the feasibility of cultivating local landraces in marginal Alpine environments, that is, in both hilly and mountain areas, to produce landrace-based foods with high nutritional value. The revival of historic rye supply chains could prove to be beneficial for the recovery process of marginal and mountain environments as well as for the preservation of the genetic heritage of rye.

## Data availability statement

The raw data supporting the conclusions of this article will be made available by the authors, without undue reservation.

## Author contributions

CS: Investigation, Formal analysis, Data curation, Writing—Original Draft; LC: Investigation, Methodology, Formal analysis, Writing—Review and Editing; MA: Investigation, Methodology, Formal analysis, Data curation, Writing—Original draft; MD: Formal analysis, Writing—Review and Editing; SRE: Investigation, Formal analysis, Writing—Review and Editing; FV: Methodology, Formal analysis, Writing—Review and Editing; ML: Conceptualization, Investigation, Writing—Review and Editing; MM: Conceptualization, Methodology, Formal analysis, Investigation, Writing—Review and Editing, Funding acquisition, Project Administration; MB: Conceptualization, Methodology, Formal analysis, Investigation, Writing—Original Draft, Funding acquisition, Supervision. All the authors have read and approved the final manuscript. All authors contributed to the article and approved the submitted version.
