# Supplementary material for: The cultivation of rye in marginal Alpine environments: a comparison of the agronomic, technological, health and sanitary traits of local landraces and commercial cultivars
Source: Front Plant Sci. 2023 May 10;14:1130543. doi: 10.3389/fpls.2023.1130543 (PMC10208067; doi:10.3389/fpls.2023.1130543)
Supplement: Supplementary file 1 [file Table_1.docx]

Supplementary Material

**Table S1.**

Rheological and mixing properties^a^ of the refined flour used as a control.

| Alveograph test | P (mm H_2_O) | 109 |
| --- | --- | --- |
|  | L (mm) | 96 |
|  | P/L | 1.14 |
|  | W (10E-4J) | 393 |
|  | Ie (%) | 65.6 |
| Farinograph test | WA (%) | 61.1 |
|  | DDT (min) | 2.3 |
|  | DS (min) | 22.5 |
|  | Degree of softening, after 10 min (FU) | 26 |
|  | Degree of softening, at 12 min after maximum (FU) | 16 |

^a^P, maximum over pressure; L, average abscissa at rupture; P/L, curve configuration ratio; W, alveographic strength; Ie, elasticity index; WA, water absorption; DDT, dough development time; DS, dough stability; FU, farinograph units.

**Table S2.**

Monthly cumulative rainfall, rainy days, and growing degree days (GDDs)^a^ measured in the experimental areas during the growth cycle in both experimental sites in the 2019-2021 period.

| **Environment** | **Month** | **Rainfall (mm)** | | **Rainy days (n°)** | | **GDDs (Ʃ °C day^-1^)** | |
| --- | --- | --- | --- | --- | --- | --- | --- |
|  |  | **2019-20** | **2020-21** | **2019-20** | **2020-21** | **2019-20** | **2020-21** |
| Hilly  (450 m) | October | 180 | 114 | 12 | 9 | 422 | 337 |
|  | November | 225 | 1 | 20 | 2 | 227 | 241 |
|  | December | 72 | 12 | 10 | 7 | 178 | 125 |
|  | January | 4 | 47 | 2 | 8 | 157 | 129 |
|  | February | 4 | 16 | 1 | 4 | 234 | 188 |
|  | March | 28 | 7 | 12 | 3 | 247 | 252 |
|  | April | 48 | 54 | 6 | 12 | 355 | 305 |
|  | May | 130 | 77 | 14 | 10 | 509 | 430 |
|  | June | 106 | 113 | 14 | 16 | 557 | 643 |
|  | July | 62 | 54 | 8 | 12 | 660 | 647 |
|  | August | 32 | 14 | 11 | 6 | 687 | 663 |
|  | **Nov-July** | **679** | **381** | **87** | **74** | **3124** | **2960** |
| Mountain (900 m) | October | 186 | 257 | 12 | 9 | 378 | 304 |
|  | November | 332 | 16 | 22 | 2 | 142 | 228 |
|  | December | 127 | 106 | 8 | 13 | 138 | 66 |
|  | January | 20 | 88 | 3 | 8 | 125 | 62 |
|  | February | 0 | 23 | 0 | 3 | 207 | 128 |
|  | March | 77 | 9 | 10 | 5 | 179 | 210 |
|  | April | 112 | 86 | 7 | 16 | 312 | 225 |
|  | May | 225 | 137 | 14 | 13 | 426 | 374 |
|  | June | 86 | 51 | 14 | 8 | 481 | 561 |
|  | July | 75 | 44 | 16 | 10 | 603 | 591 |
|  | August | 42 | 19 | 13 | 9 | 630 | 611 |
|  | **Nov-July** | **1054** | **560** | **94** | **78** | **2613** | **2445** |

^a^Accumulated growing degree days for each experiment using a 0 °C base value. Data obtained from the ARPA Piemonte agrometeorological service.

**Table S3.**

Effect of the genotype, the harvest year and their interaction on the agronomic traits of the investigated wheat and rye cultivars, cultivated in two different marginal Alpine environments.

| **Environment** | **Factors** | **Source of variation** | **Full flowering**  **(days from the 1^st^ of April)** | | **Flowering duration (days)** | | **Plant height (cm)** | | **Culm diameter (mm)** | |
| --- | --- | --- | --- | --- | --- | --- | --- | --- | --- | --- |
| Hilly  (450 m) | **Genotype (G)** | Bread wheat | 41 | b | 7 | c | 76 | d | 3.7 | d |
|  |  | Susa rye | 46 | a | 13 | b | 145 | bc | 4.1 | bc |
|  |  | Maira rye | 45 | a | 17 | a | 161 | a | 3.9 | cd |
|  |  | Tanaro rye | 45 | a | 13 | b | 144 | bc | 4.5 | ab |
|  |  | Commercial rye | 46 | a | 12 | b | 149 | b | 4.4 | ab |
|  |  | Hybrid rye | 44 | a | 12 | b | 138 | c | 4.7 | a |
|  |  | *p* (F) | *** | | *** | | *** | | *** | |
|  | **Year (Y)** | 2020 | 42 | b | 11 | b | 132 | b | 4.1 | b |
|  |  | 2021 | 47 | a | 13 | a | 139 | a | 4.4 | a |
|  |  | *p* (F) | *** | | *** | | *** | | ** | |
|  | **G × Y** | *p* (F) | *** | | ns | | ** | | ns | |
| Mountain  (900 m) | **Genotype (G)** | Bread wheat | 60 | a | 8 | c | 81 | d | 3.4 | c |
|  |  | Susa rye | 60 | a | 10 | b | 149 | b | 3.8 | ab |
|  |  | Maira rye | 61 | a | 12 | a | 177 | a | 3.5 | bc |
|  |  | Tanaro rye | 61 | a | 11 | b | 148 | b | 3.9 | ab |
|  |  | Commercial rye | 61 | a | 10 | b | 154 | b | 3.8 | ab |
|  |  | Hybrid rye | 57 | b | 11 | b | 139 | c | 4.0 | a |
|  |  | *p* (F) | *** | | *** | | *** | | *** | |
|  | **Year (Y)** | 2020 | 60 | a | 10 | b | 135 | b | 3.3 | b |
|  |  | 2021 | 60 | a | 11 | a | 148 | a | 4.2 | a |
|  |  | *p* (F) | ns | | *** | | *** | | *** | |
|  | **G × Y** | *p* (F) | * | | *** | | ns | | ns | |

Means followed by different letters are significantly different, according to the REGW-F test [(*) *p* (F) ≤ 0.05, (**) *p* (F) ≤ 0.01, (***) *p* (F) ≤ 0.001, and ns, non-significant].

**Table S4.**

Pearson correlation coefficients of the agronomic (A), nutritional and phytochemical (B), and qualitative and rheological (C) traits^a^.

(A)

| **Traits** | Ear density | | Kernels ear^-1^ | | TKW | | Straw yield | | HI | | Full flowering | | Flowering duration | | Plant height | | Culm diameter | |
| --- | --- | --- | --- | --- | --- | --- | --- | --- | --- | --- | --- | --- | --- | --- | --- | --- | --- | --- |
| GY | -0.011 |  | 0.250 | * | 0.206 |  | 0.169 |  | 0.591 | ** | -0.027 |  | -0.536 | ** | -0.197 |  | -0.433 | ** |
| Ear density |  |  | -0.410 | ** | 0.267 | * | 0.118 |  | -0.168 |  | -0.175 |  | 0.073 |  | -0.315 | ** | -0.399 | ** |
| Kernels ear^-1^ |  |  |  |  | -0.381 | ** | -0.198 |  | -0.328 | ** | 0.459 | ** | -0.362 | ** | 0.565 | ** | 0.637 | ** |
| TKW |  |  |  |  |  |  | 0.129 |  | 0.078 |  | -0.582 | ** | 0.012 |  | -0.568 | ** | -0.256 | * |
| Straw yield |  |  |  |  |  |  |  |  | -0.670 | ** | 0.064 |  | -0.066 |  | 0.053 |  | -0.203 |  |
| HI |  |  |  |  |  |  |  |  |  |  | -0.117 |  | -0.310 | ** | -0.213 |  | -0.510 | ** |
| FFD |  |  |  |  |  |  |  |  |  |  |  |  | -0.394 | ** | 0.659 | ** | 0.382 | ** |
| FD |  |  |  |  |  |  |  |  |  |  |  |  |  |  | 0.089 |  | -0.543 | ** |
| Plant height |  |  |  |  |  |  |  |  |  |  |  |  |  |  |  |  | 0.260 | * |

(B)

| **Traits** | TAX | | β-glucans | | SPAs | | CWBPAs | | AC_ABTS_ | | AC_FRAP_ | |
| --- | --- | --- | --- | --- | --- | --- | --- | --- | --- | --- | --- | --- |
| TKW | -0.510 | ** | -0.519 | ** | -0.389 | ** | 0.163 |  | -0.446 | ** | -0.442 | ** |
| TAX |  |  | 0.847 | ** | 0.444 | ** | -0.026 |  | 0.704 | ** | 0.676 | ** |
| β-glucans |  |  |  |  | 0.513 | ** | -0.045 |  | 0.821 | ** | 0.705 | ** |
| SPAs |  |  |  |  |  |  | 0.466 | ** | 0.507 | ** | 0.361 | ** |
| CWBPAs |  |  |  |  |  |  |  |  | 0.061 |  | 0.016 |  |
| AC_ABTS_ |  |  |  |  |  |  |  |  |  |  | 0.699 | ** |

(C)

| **Traits** | GPC | | Ash | | TAX | | β-glucans | | DDT | | DS | | C1 | | C1-C2 | | C3-C2 | | C3-C4 | | C5-C4 | |
| --- | --- | --- | --- | --- | --- | --- | --- | --- | --- | --- | --- | --- | --- | --- | --- | --- | --- | --- | --- | --- | --- | --- |
| TKW | 0.149 |  | -0.319 | ** | -0.510 | ** | -0.519 | ** | 0.560 | ** | 0.509 | ** | -0.514 | ** | -0.196 |  | 0.249 | * | -0.328 | ** | -0.022 |  |
| GPC |  |  | 0.298 | * | -0.342 | ** | -0.489 | ** | 0.342 | ** | 0.232 |  | -0.290 | * | 0.283 | * | 0.005 |  | 0.011 |  | -0.253 | * |
| Ash |  |  |  |  | 0.267 | * | 0.186 |  | -0.274 | * | -0.250 | * | 0.375 | ** | 0.433 | ** | 0.047 | ** | 0.281 | * | 0.029 |  |
| TAX |  |  |  |  |  |  | 0.847 | ** | -0.759 | ** | -0.566 | ** | 0.414 | ** | -0.111 |  | -0.450 | ** | 0.548 | ** | -0.112 |  |
| β-glucans |  |  |  |  |  |  |  |  | -0.817 | ** | -0.601 | ** | 0.392 | ** | -0.814 |  | -0.574 | ** | 0.617 | ** | -0.815 |  |
| DDT |  |  |  |  |  |  |  |  |  |  | 0.758 | ** | -0.521 | ** | -0.041 |  | 0.126 |  | -0.690 | ** | -0.106 |  |
| DS |  |  |  |  |  |  |  |  |  |  |  |  | -0.345 | ** | -0.060 |  | -0.285 | ** | -0.646 | ** | -0.302 | ** |
| C1 |  |  |  |  |  |  |  |  |  |  |  |  |  |  | 0.385 | ** | 0.362 | ** | -0.004 |  | 0.548 | ** |
| C1-C2 |  |  |  |  |  |  |  |  |  |  |  |  |  |  |  |  | 0.075 |  | -0.004 |  | -0.090 |  |
| C3-C2 |  |  |  |  |  |  |  |  |  |  |  |  |  |  |  |  |  |  | -0.377 | ** | 0.827 | ** |
| C3-C4 |  |  |  |  |  |  |  |  |  |  |  |  |  |  |  |  |  |  |  |  | -0.367 | ** |
| C5-C4 |  |  |  |  |  |  |  |  |  |  |  |  |  |  |  |  |  |  |  |  |  |  |

^a^GY, grain yield; TKW, thousand kernel weight; HI, harvest index; TAX, total arabinoxylans; SPAs, soluble phenolic acids; CWBPAs, cell wall-bound phenolic acids; AC, antioxidant capacity (ABTS and FRAP assays); GPC, grain protein content; DDT, dough development time; DS, dough stability; C1, peak of 1.1 Nm ± 0.05 at 30 °C; C1-C2, thermal weakening; C3-C2, gelatinization; C3-C4, cooking stability range; C5-C4, cooling setback. Marked factors are statistically significant at (*) *p* < 0.05, and (**) *p* < 0.01.
